# Examining Dyadic Stress Appraisal Processes Within Romantic Relationships from a Challenge and Threat Perspective

**DOI:** 10.1007/s42761-024-00235-3

**Published:** 2024-01-30

**Authors:** Brett J. Peters, Nickola C. Overall, Abriana M. Gresham, Ashley Tudder, Valerie T. Chang, Harry T. Reis, Jeremy P. Jamieson

**Affiliations:** 1 Ohio University, Athens, OH USA; 2 University of Auckland, Auckland, New Zealand; 3 Washington University in St. Louis, St. Louis, MO USA; 4 University of Rochester, Rochester, NY USA

**Keywords:** Stress, Appraisals, Close relationships, Dyadic data analysis

## Abstract

The biopsychosocial model of challenge and threat emphasizes how individuals appraise stress. Close relationship theories emphasize the interpersonal context, communication, and outcomes that arise from stress. We integrate these approaches by examining the individual variability surrounding appraisals of sufficient (more challenge, less threat) or insufficient (more threat, less challenge) resources to cope with demands and examining how these appraisals are associated with couples’ behavior and feelings toward each other. Across three studies, 459 romantic couples (*N* = 918), and various potentially stressful in-lab conversations (extra-dyadic problem, dislikes about each other, dependability, and relationship conflict), we found evidence that stress appraisals indicative of more challenge and less threat were associated with more approach- and less avoidance-oriented behaviors within interactions. These approach- and avoidance-oriented behaviors were associated with greater feelings of relationship security and well-being after the conversation. However, whose (actors or partners) appraisals and behaviors were associated with security and well-being varied across the three studies. This work provides theoretical and empirical evidence for an interpersonal emphasis on intraindividual stress appraisal processes through a dyadic and close relationships lens. Our integrative theoretical framework breaks away from the idea that stress is inherently “bad” or “maladaptive” to show that appraising stress as more manageable (more challenge, less threat) is associated with more relationship behaviors that approach incentives and less that avoid threats and enhance feelings of relationship security and well-being.

**Supplementary Information:**

The online version contains supplementary material available at 10.1007/s42761-024-00235-3.

Stress is the anticipation or experience of encountering demands in goal-related contexts where the outcome is not implied nor necessarily negative (Crum et al., 2020). According to the biopsychosocial model of challenge and threat, when faced with a stimulus that could cause stress, cognitive and affective appraisals of demands (e.g., uncertainty, danger) relative to coping resources (e.g., skills/ability, familiarity) can determine affect, motivation, and behavior (e.g., Blascovich, 2013; Jamieson, 2017; Jamieson & Elliot, 2018). When individuals perceive sufficient resources to cope with demands, they exhibit greater challenge and less threat and enact more approach-oriented behaviors, whereas when appraisals of demands outweigh resources, individuals exhibit greater threat and less challenge and enact more avoidance-oriented behaviors (Blascovich, 2013; Jamieson & Elliot, 2018). The biopsychosocial model of challenge and threat places emphasis on cognitive and affective stress appraisals which allows for a nuanced understanding of stress (that stress is not necessarily “bad”), but often ignores the interpersonal contexts in which stressors tend to unfold.

Separately, a corpus of research has examined stress within close relationships. Central theoretical models emphasize how stress is communicated and influences key interpersonal behaviors and relationship outcomes (Bodenmann et al., 2015; Falconier & Kuhn, 2019; Lavee, 2013), whether the stress is internal or external to the relationship (Buck & Neff, 2012; Neff & Karney, 2004), and that certain interpersonal contexts may trigger stress (Karney & Bradbury, 1995) or response systems, such as the attachment system (Simpson & Rholes, 2017). However, the nuanced assessments of stress appraisals that are present in stress models tend to be absent from close relationship models of stress (cf., Falconier & Kuhn, 2019). Stress theories like the biopsychosocial model of challenge and threat focus on how an individual is appraising stress, whereas relationship theories emphasize the interpersonal context, communication, and outcomes that arise from stress. Though both perspectives lend valuable insight, they are not well integrated (cf., Falconier & Kuhn, 2019).

The goal of the current research is to integrate the strengths of these two approaches by examining 1) the individual variability surrounding whether stress is appraised as more challenging or threatening, and 2) examining how these appraisals are associated with both individuals’ and their partners’ behavior and feelings toward each other. For example, stress arising from negotiating finances within a relationship may be appraised as something that is more manageable (more challenge, less threat) for one dyad member, but less so (more threat, less challenge) for the other. By assessing stress appraisals dyadically (Kenny et al., 2006), we can examine how both individuals’ appraisal processes are associated with each other’s behavior and feelings of the relationship. Specifically, we contend that the way each dyad member appraises stress can shape how they behave toward each other and impact feelings of relationship security and well-being.

To determine what kinds of relationship behaviors may be associated with individuals’ stress appraisals, we integrated approach and avoidance orientation processes that undergird challenge and threat stress responses (Blascovich, 2013; Jamieson & Elliot, 2018), with general motives within interpersonal relationships to guide individuals toward favorable relationship outcomes (approach) or away from potential threats (avoidance; Elliot, 1999; Gable & Gosnell, 2013; Gable & Impett, 2012; Impett et al., 2010; Naragon-Gainey et al., 2017). Relationship behaviors that result from more approach-oriented challenge (and less threat) responses—referred to henceforth as approach-oriented behaviors—include reassuring partners, conveying commitment and trust, engaging partners in cooperative conflict resolution, and ensuring smooth communication (Gable & Gosnell, 2013; Gable & Impett, 2012; Jamieson & Elliot, 2018; Overall & McNulty, 2017; Overall, Maner et al., 2022; Overall, Pietromonaco et al., 2022). When experiencing more threat and less challenge, avoidance-oriented behaviors include trying to not hurt a partner’s feelings, avoiding disagreement, and hiding negative thoughts (e.g., Gable & Impett, 2012; Jamieson & Elliot, 2018; Overall & McNulty, 2017; Overall, Maner et al., 2022; Overall, Pietromonaco et al., 2022).

Our dyadic approach to stress appraisals also suggests that partners’ appraisals of stress may influence individuals’ behaviors within interactions. Relative to intraindividual actor effects, we suspect interpersonal partner effects to be more tenuous, as various literatures suggest several potential mechanisms through which partners’ appraisals could influence individuals’ behavior, which may not necessarily operate consistently across individuals, conversation contexts, and relationships. These partner effects may be more contingent on how stress is communicated (e.g., Bodenmann et al., 2015; Falconier & Kuhn, 2019), how affect is expressed (Oveis et al., 2020), prior knowledge of partners’ personality traits or responses to specific stressors (Lemay & Clark, 2008; Pietromonaco & Overall, 2022), or broader perceptions of emotional capital and commitment (Arriaga et al., 2006; Feeney & Lemay, 2012).

The central aim of the current research was to integrate the intraindividual stress appraisal processes emphasized by the biopsychosocial model of challenge and threat with a dyadic framework highlighted by relationship theories. We tested whether the way individuals appraised their stress was associated with the types of behaviors they engaged in with their partners (H1) and whether these appraisals (H2) and behaviors (H3) were associated with feelings of relationship security and quality. Table 1 summarizes operationalizations of each construct across the three studies. Individuals who (actor effect, H1a), and individuals whose partners (partner effect, H1p), appraise a potential stressor as being more challenging and less threatening will enact more approach-oriented and less avoidance-oriented behaviors within couple interactions. Individuals who (H2a), and individuals whose partners (H2p), appraise potential stressors as more challenging and less threatening and enact more approach-oriented and less avoidance-oriented behavior (H3a and H3p) will foster more positive feelings of relationship security and well-being after the conversation.

**Table 1:** Operationalizations of key constructs across studies 1, 2, and 3

Study and site	Context	Stress appraisals	Behavior within interactions	Feelings of relationship security and well-being
Study 1 Ohio University	Discussion about extra-dyadic problem	Resource/demand appraisals	Observed positive-indirect behaviors	Responsiveness, intimacy, and closeness
Study 2 University of Rochester	Potentially stressful relationship-oriented discussion	Resource/demand appraisals	Self-reported approach/avoidance behaviors	Responsiveness, intimacy, and closeness
Study 3 University of Auckland	Relationship conflict discussion	Resource/demand appraisals	Self-reported approach/avoidance conflict behaviors	Responsiveness, intimacy, and closeness

## Study 1

In study 1, couples discussed their most significant, ongoing problem that did not explicitly involve their significant other. Stress external to the relationship can cause problems within the relationship and thus is an important context to examine how appraisals of stress within these conversations influence relationship behavior and well-being (Buck & Neff, 2012; Neff & Karney, 2004; Randall & Bodenmann, 2009).

### Method

#### Power

As part of a larger project examining co-rumination in close relationships (see Gresham et al., 2023), a series of Monte Carlo simulations were conducted with equality constraints for the paths modeling effects of each dyad (Lane & Hennes, 2018). Past dyadic datasets with similar measures were used to approximate effects with respect to physiological assessments (Peters & Jamieson, 2016; Peters et al., 2014, 2018, 2019). Based on hypothesized small-to-medium interaction effects (*r* ~ 0.15) and consistent with past observed effect sizes, it was determined that 120 dyads would be needed to achieve sufficient power (> .90) for the hypotheses proposed as part of the larger study. The a priori power analyses and original hypotheses (unrelated to the current aims) involved actor and partner three-way interaction effects. This provides confidence that study 1 is adequately powered for the hypothesized main effects within an actor-partner interdependence model (APIM).

#### Participants

Participants were recruited through Ohio University’s SONA system, campus-wide e-mails, and flyers. Before participation, interested couples were screened for exclusion (e.g., presence of a cardiac pacemaker, diagnosis of hypertension, medications with cardiac side effects) and inclusion criteria (e.g., being at least 18 years old and in a romantic relationship together for at least 3 months). A total sample of 280 individuals (140 dyads) was recruited. One dyad was excluded after disclosing they did not meet inclusion criteria, leaving a final sample of 139 dyads (*N* = 278). Participants were primarily White (*N* = 235), non-Hispanic (*N* = 264), and had an average age of 20.20 years (*SD* = 2.49) and relationship length of 22.43 months (*SD* = 22.49). Dyads were primarily composed of different genders (*N* = 125 dyads, 91.9%; 131 man/male, 138 woman/female, 2 other, 7 participants failed to report their gender). See Table S1 for full sample characteristics.

#### Procedure

Study procedures were approved by Ohio University’s institutional review board. Couples arrived at the lab together and were separated into private testing rooms where they completed an initial intake interview and questionnaires. Each couple member identified and ranked two personal problems that did not include their romantic partner. Couple members were then randomly assigned to roles for a problem discussion task: the *discloser* (who would share an ongoing stressful problem) and the *responder* (who would respond to their partner’s [discloser’s] problem). The most stressful and important problem for the discloser was chosen by the experimenter to use as the topic for the problem discussion task. Experimenters informed each participant that they would discuss the discloser’s problem. Because the initial aim of this study was to examine co-rumination in romantic relationships and included an experimental manipulation of co-rumination, participants were given different instructions based on their condition assignment (co-ruminate during the upcoming conversation vs. talk about the problem naturally). A full overview of the co-rumination manipulation is presented in the OSM.

Participants were given three minutes to gather their thoughts on the topic and after they reported on their stress appraisals regarding the upcoming conversation. Following the 3-min anticipation period, the wall separating the two private testing rooms was collapsed and the participants engaged in an 8-min face-to-face conversation concerning the discloser’s problem. After the end of the problem discussion, the wall was re-extended for privacy and participants answered an additional set of questionnaires. After completion, participants were debriefed and compensated with either a USD$16 e-gift card or 2 h of course credit.

#### Measures

Full versions of all self-report scales and behavioral coding schedules across studies 1, 2, and 3 are included in the OSM.

##### Stress Appraisals

Immediately before the conversation, participants completed a 4-item questionnaire on 7-point Likert scales (1 = strongly disagree, 7 = strongly agree) that assessed their appraisals of resources and demands of the upcoming conversation with their partner (Beltzer et al., 2014; Mendes et al., 2007). Two items assessed appraisals of resources (e.g., *I have the abilities to do well during the conversation*; *α* = .714) and two items assessed appraisals of demands (e.g., *The upcoming conversation is very demanding*; *α* = .814). A challenge-threat index was created by subtracting demands from resources such that higher scores indicated more challenge and less threat (Behnke & Kaczmarek, 2018; Beltzer et al., 2014; Mendes et al., 2007; Seery, 2011).

##### Behavior Within Interactions

Two coders blind to condition assignment and hypotheses independently coded behaviors during the conversation using a coding scheme that characterizes the breadth and depth of communication behaviors in romantic relationships (Overall & McNulty, 2017; Overall et al., 2009; Tudder et al., 2020). Pre-registered analyses (10.17605/OSF.IO/KCAQN) focused on one behavior from this coding scheme that most closely aligned with approach-oriented behaviors. Positive-indirect (or loyalty) behaviors were defined as behaviors that emphasize validating partners’ point of view, acknowledging the importance of the topic, reassuring partners that they can get through the stressor together, and being optimistic. The more approach-oriented aspects of these behaviors were also more relevant and more closely aligned to the behaviors typically examined in the context of couples’ discussions of personal problems. Coders considered frequency, intensity, and duration of behaviors across the first (ICC = .863, *M* = 1.52, *SD* = 0.74) and second (ICC = .831, *M* = 1.45, *SD* = 0.69) halves of the conversation for each individual in the dyad (1 = low, 3 = moderate, 5 = high). Coder ratings across both halves of the conversation were combined to form an overall approach-oriented behavior score (*M* = 1.48, *SD* = 0.66).

##### Feelings of Relationship Security and Well-Being

To assess feelings of relationship security and well-being, we targeted key relationship processes of perceived partner responsiveness and feelings of intimacy and closeness (Reis & Shaver, 1988; Reis et al., 2004) with seven items that asked the extent to which participants agreed or disagreed that they, for example, felt, “close/intimate with [their] partner,” “understood/validated by [their] partner,” (*α* = 0.894) on 7-point Likert scales (1 = not at all, 7 = very much).

##### Baseline Assessments of Relationship Satisfaction

After the consent process and during the initial battery of questionnaires, participants completed the 16-item couples satisfaction index (Funk & Rogge, 2007) to assess baseline levels of relationship satisfaction (e.g., my relationship with my partner makes me happy) on 6- and 7-point Likert scales (e.g., 0 = not at all true, 5 = completely true, *α* = .931). Relationship satisfaction was controlled for in ancillary analyses described below.

### Results

See Table 2 for the descriptive statistics and zero-order correlations between primary study measures. We followed the guidelines and associated SPSS syntax provided by Kenny and colleagues (Kenny et al., 2006) to run a series of APIMs that accounted for the dependence associated across partners. When applicable and across all three studies, effect size (*r*) was estimated using the following formula: *r* = √(*t*^2^ / (*t*^2^ + *df*)).

**Table 2:** Descriptive statistics and zero-order correlations among study 1 variables

	*M (SD)*	1	2	3
1. Actors’ stress appraisals	2.56 (2.04)	-		
2. Actors’ positive-indirect behaviors	1.48 (0.66)	.191**	-	
3. Actors’ perceived responsiveness, intimacy, and closeness	6.65 (0.52)	.170**	.058	-
4. Partners’ stress appraisals	2.56 (2.04)	.219**	.046	.069
5. Partners’ positive-indirect behaviors	1.48 (0.66)	.046	.084	.104

** = *p* < .01

#### Stress Appraisals in Anticipation of a Conversation on Positive-Indirect Behaviors During the Conversation (H1)

In support of H1a but not H1p, as shown in Table 3, when actors (but not partners) anticipated the upcoming conversation as more challenging and less threatening, actors engaged in more positive-indirect behaviors.

**Table 3:** The associations between actors’ and partners’ anticipatory stress appraisals and positive-indirect behaviors during the conversation (study 1, hypothesis 1)

	Positive-indirect behaviors				
Predictor variables	*B*	*SE*	*t*	*p*	*r*
Actors’ stress appraisals	0.06	0.02	3.18	.002	.21
Partners’ stress appraisals	0.01	0.02	0.31	.756	.02

#### Stress Appraisals (H2) and Positive-Indirect Behaviors (H3) on Feelings of Relationship Security and Well-Being

In support of H2a but not H2p (see Table 4), actors (but not partners) who appraised the conversation as more challenging and less threatening perceived their partner as more responsive and felt closer and more intimate with their partner after the conversation. Actors’ (H3a) and partners’ (H3p) positive-indirect behaviors were not associated with actors’ perceived responsiveness or feelings of closeness and intimacy.

**Table 4:** The associations between actors’ and partners’ stress appraisals (hypothesis 2) and positive-indirect behaviors (hypothesis 3) on responsiveness, intimacy, and closeness (study 1)

	Responsiveness, intimacy, and closeness				
Predictor variables	*B*	*SE*	*t*	*p*	*r*
Actors’ stress appraisals	0.04	0.02	2.46	.015	.16
Actors’ positive-indirect behaviors	0.04	0.05	0.77	.444	.05
Partners’ stress appraisals	0.01	0.02	0.58	.561	.04
Partners’ positive-indirect behaviors	0.06	0.05	1.08	.279	.07

#### Ancillary Analyses

We conducted follow-up analyses to account for potential confounding variables, including experimental condition (co-rumination vs. natural control condition), conversation role assignments (discloser of extra-dyadic problem vs. responder to the problem), and baseline assessments of actors’ and partners’ relationship satisfaction. All effects reported in the main pre-registered analyses for H1a remained significant. However, actors’ greater baseline relationship satisfaction was significantly associated with greater perceived responsiveness, closeness, and intimacy after the conversation (*B* = 0.05, *SE* = 0.003, *t* = 16.57, *p* < .001, *r* = .79), and these strong associations weakened the main effect of actors’ stress appraisals on closeness and intimacy, which was no longer significant (H2a, *B* = 0.02, *SE* = 0.012, *t* = 1.44, *p* = .152, *r* = .10). Separate analyses including experimental condition, conversation role, and all possible interactions did not alter the main findings for tests of hypotheses 2 and 3. See OSM for a presentation of the full model and a description of significant effects not germane to the current investigation. During the review process, we also conducted additional exploratory analyses (see OSM for a summary). The pattern of results did not change when controlling for and examining potential moderation effects of self-report items that assessed actor and partner stress intensity. Moreover, we did not observe interactions between actor’s and partner’s stress appraisals on behavior or feelings of relationship security and well-being.

## Study 2

With some initial support for hypotheses 1 and 2 (but not 3), in study 2 we developed a new questionnaire that assessed our conceptualization of approach- and avoidance-oriented relationship behaviors and tested hypotheses within a potentially stressful context.

### Method

#### Power

The data reported here were part of a larger project examining perceptions of attachment insecurities (Peters et al.,^ 2017^). A series of Monte Carlo simulations were conducted with equality constraints for the paths modeling effects of each dyad (Lane & Hennes, 2018). Focusing on the hypothesized interaction effects from this larger project with a small to medium effect size (*r* ~ .20), approximately 160 dyads were needed for sufficient power (> .90). To account for potential video equipment malfunctions and inattentiveness of participants, once 160 dyads was reached, data collection continued until the end of the academic semester.

#### Participants

Participants were recruited through the University of Rochester’s SONA system and flyers. As in study 1, participants had to be in a romantic relationship with each other for at least 3 months. A total sample of 354 individuals in 177 dyads participated in the study. Individuals (*N* = 14) were excluded from analyses if they failed to correctly answer attention-check items. Participants were primarily White (*N* = 192), non-Hispanic (*N* = 315), and had an average age of 20.20 years (*SD* = 1.87) and relationship length of 15.57 months (*SD* = 11.99). Dyads were primarily composed of different genders (*N* = 169 dyads, 95%; 171 men/males, 183 women/females). See Table S1 for full sample characteristics.

#### Procedure

Upon arrival, dyad members were separated into private testing rooms where they provided consent and completed initial questionnaires. Couples were randomly assigned to one of two 8-min discussions: one about the things they disliked most (pet-peeves) about each other (*N* = 89; see OSM for full manipulation instructions) or the top three ways in which they depend on their partners (*N* = 88). These prompts targeted key working models of security and related fears in relationships (i.e., fears of rejection and dependence). Participants then completed a preparation period during which they were given three minutes to “gather their thoughts” for the conversation and completed a battery of questionnaires. Participants were then brought together to engage in the conversation. Finally, participants returned to their private testing rooms and relaxed for a 3-min recovery period and filled out additional questionnaires. Participants were compensated with 2 h of credit or USD$10–20.

### Measures

#### Stress Appraisals

As in study 1, four items assessed resource (*α* = 0.850) and demand (*α* = .754) appraisals. Demand appraisals were subtracted from resource appraisals to form a challenge and threat index.

#### Behavior Within Interactions

To assess approach- and avoidance-oriented behaviors, we created a new scale that identified key approach- and avoidance-related behaviors important for couples’ communication outcomes (Overall & Simpson, 2015; Simpson & Overall, 2014). Six items assessed approach-oriented behaviors (e.g., *I reassured my partner that everything was going to be okay*; *α* = .770) and five assessed avoidance-oriented behaviors (e.g., *I downplayed the severity of the problem; α* = .773) during the conversation. A parallel analysis was conducted with 5000 parallel data sets that reshuffled the original data to remove the correlations but retained the distributions (means, *SD*, skew). The results provided statistical justification for the expected two-component solution. See the OSM for the full scale.

#### Feelings of Relationship Security and Well-Being

Study 2 included separate assessments of perceived responsiveness and closeness and intimacy. Participants rated 12 items assessing their partner’s responsiveness (Reis et al., 2017; *α* = .976, *…saw the “real” me, understood me, was responsive to my needs*; 1 = not at all true, 7 = completely true). Closeness and intimacy were assessed with two face-valid items (*Do you feel closer with your partner, do you feel more intimate with your partner*, *α* = .934; 1 = not at all, 7 = very much). These two composites were standardized and combined to form a responsiveness, closeness, and intimacy composite.

#### Baseline Assessments of Relationship Satisfaction

During the initial battery of questionnaires after the consent process, participants completed the 16-item couples satisfaction index (Funk & Rogge, 2007; *M* = 68.34, *SD* = 11.34, *α* = .939). Relationship satisfaction was controlled for in ancillary analyses described below.

### Results

Descriptive statistics and zero-order correlations between primary study measures are presented in Table 5. We followed the guidelines and associated SPSS syntax provided by Kenny and colleagues (Kenny et al., 2006) to run a series of APIMs that accounted for the dependence associated across partners. Dyads were distinguished by gender, with same-gender couples (*N* = 8) randomly assigned on the distinguishing variable for analyses. Note, additional analyses were conducted in which same-sex couples were removed. All effects reported remained significant.

**Table 5:** Descriptive statistics and zero-order correlations among primary study variables

	*M* (SD)	1	2	3	4	5
1. Actors’ stress appraisals	1.17 (2.69)	-				
2. Actors’ approach behaviors	5.45 (0.93)	.277	-			
3. Actors’ avoidance behaviors	2.80 (1.23)	− .259	− .051^	-		
4. Actors’ responsiveness, intimacy, and closeness	0.00 (2.69)	.335	.476	− .321	-	
5. Partners’ stress appraisals	1.17 (2.69)	.213	.189	− .166	.318	-
6. Partners’ approach behaviors	5.45 (0.93)	.189	.114	− .042^	.234	.277
7. Partners’ avoidance behaviors	2.80 (1.23)	− .166	− .42^	.298	− .141	− .259

All correlations are significant at *p* < .05, except for those denoted with ^*.* Responsiveness and intimacy and closeness composites were standardized and combined; thus the mean is zero

#### Stress Appraisals in Anticipation of a Conversation and Approach and Avoidance Behaviors During the Conversation (H1)

Approach and avoidance behaviors (in separate analyses) were regressed on actors’ and partners’ stress appraisals. As shown in Table 6, when actors and partners anticipated the upcoming conversation as more challenging and less threatening, actors engaged in more approach and less avoidance behaviors, supporting H1a and H1p.

**Table 6:** The associations between actors’ and partners’ anticipatory stress appraisals and approach and avoidance behaviors during the conversation (study 2, hypothesis 1)

	Approach behaviors					Avoidance behaviors				
Predictor variables	*B*	*SE*	*t*	*p*	*r*	*B*	*SE*	*t*	*p*	*r*
Actors’ stress appraisals	0.08	0.02	4.37	< .001	.24	− 0.11	0.02	− 4.54	< .001	.25
Partners’ stress appraisals	0.05	0.02	2.58	.010	.15	− 0.05	0.02	− 2.24	.026	.12

#### Associations Between Stress Appraisals (H2) and Behavior (H3) on Feelings of Relationship Security and Well-Being

The results shown in Table 7 support hypothesis 2. Actors (H2a) and partners (H2p) who appraised the conversation as more challenging and less threatening perceived their partner as more responsive and felt more intimate and closer after the conversation.

**Table 7:** The associations between actors’ and partners’ stress appraisals (hypothesis 2) and approach and avoidance behaviors (hypothesis 3) during the conversation on responsiveness, intimacy, and closeness (study 2)

	Responsiveness, intimacy, and closeness				
Predictor variables	*B*	*SE*	*t*	*p*	*r*
Actors’ stress appraisals	0.04	0.02	2.75	.006	.15
Actors’ avoidance behaviors	− 0.16	0.03	− 5.02	< .001	.28
Actors’ approach behaviors	0.35	0.04	8.31	< .001	.43
Partners’ stress appraisals	0.05	0.02	3.11	.002	.17
Partners’ avoidance behaviors	0.01	0.03	0.34	.731	.02
Partners’ approach behaviors	0.11	0.04	2.51	.013	.14

For hypothesis 3, actors (H3a) and partners (H3p) who engaged in more approach behaviors during the conversation perceived their partners as more responsive and felt more intimate and closer with their partners. In contrast, actors (but not partners) who engaged in more avoidance behaviors perceived their partners less positively and felt less close and intimate with their partners.

#### Ancillary Analyses

Like study 1, findings in support of H1a, H1p, H3a, and H3p generally remained significant when controlling for potential confounds of experimental condition, role in the conversation, and relationship satisfaction. Findings for H2a (but not H2p) were attenuated when controlling for relationship satisfaction (see OSM). During the review process, we also conducted additional exploratory analyses (see OSM for a summary). The pattern of results did not change when controlling for and examining potential moderation effects of self-report items that assessed actor and partner stress intensity. Moreover, we did not observe interactions between actor’s and partner’s stress appraisals on behavior or feelings of relationship security and well-being.

## Study 3

In study 3, couples discussed their most significant, ongoing relationship issue. Conflict discussions represent times when relationships are tested (Cavallo et al., 2013; Kelley & Thibaut, 1978) and are a potent form of stress (Broderick, 1981; Papp et al., 2009; Storaasli & Markman, 1990) in which responses have direct repercussions for relationship wellbeing (Karney & Bradbury, 1995; Le et al., 2010; Rusbult & Van Lange, 2008).

### Method

#### Power

We used an archival dataset to test our theoretical model. The a priori power analysis reported elsewhere (Chang & Overall, 2022; Overall, Maner et al., 2022; Overall, Pietromonaco et al., 2022) suggested the sample size provided > .90 power for small-medium actor effects (*r* = .20) when controlling for typical dependence across couple members (Ackerman et al., 2016).‬‬‬‬‬‬‬‬‬‬‬‬‬‬‬‬‬‬‬‬‬‬

#### Participants

As part of the larger project examining emotion regulation and perceived stress during couples’ conflict, 280 participants in 140 dyads were targeted (Chang & Overall, 2022; Overall, Maner et al., 2022; Overall, Pietromonaco et al., 2022). Paper and electronic advertisements were posted across university-based organizations and social-media-invited people in long-term relationships (at least one year) to participate. Two-hundred and eighty-six individuals (143 dyads) were recruited. Participants were primarily New Zealand European/Pākehā (38.7%) or Asian (25.7%) and had an average age of 24.73 years (*SD* = 7.10) and relationship length of 41.52 months (*SD* = 51.24). Dyads were primarily composed of different genders (*N* = 138 dyads, 97%). See Table S1 for full sample characteristics.

#### Procedure

Upon arrival, dyad members provided consent and completed initial questionnaires. Next, they ranked in order of significance three serious relationship issues that caused conflict within their relationship. One of the couple members was randomly chosen before they arrived at the lab to discuss their most serious and ongoing issue with their partner. Before and after the conversation, participants were given a battery of questionnaires (see Measures). Participants received NZ$100.

#### Measures

All items were assessed on a 7-point Likert scale from 1 (not at all) to 7 (very much). Full scales are included in the OSM.

##### Stress Appraisals

As in studies 1 and 2, four items were used to assess resource (*α* = .754) and demand (*α* = .779) appraisals. Items were adjusted to refer directly to the conflict context (e.g., study 3: *I am able to do the things needed to settle this issue*; studies 1 and 2*: I have the abilities to do well during the conversation*). Demand appraisals were subtracted from resource appraisals to form a challenge and threat appraisal index.

##### Behavior Within Interactions

To assess approach and avoidance behaviors, we used the same scales from study 2 but modified and added some items to refer directly to the conflict context (see OSM). Seven items assessed approach-oriented behaviors (*α* = .882) and 7 items assessed avoidance-oriented behaviors (*α* = .869) during the conversation.

##### Feelings of Relationship Security and Well-Being

Responsiveness, intimacy, and closeness were assessed with the same seven items used in study 1 (*α* = .960).

##### Baseline Assessments of Relationship Satisfaction

During the initial battery of questionnaires after the consent process, participants completed five items assessing relationship satisfaction on 7-point Likert scales (e.g., *I feel satisfied with our relationship*; *M* = 6.08, *SD* = 0.81, *α* = .838). Relationship satisfaction was controlled for in ancillary analyses described below.

### Results

Descriptive statistics and zero-order correlations between primary study measures are presented in Table 8. We followed the guidelines and associated SPSS syntax provided by Kenny and colleagues (Kenny et al., 2006) to run a series of APIMs that accounted for the dependence associated across partners. Analyses were preregistered (10.17605/OSF.IO/WRGJH).

**Table 8:** Descriptive statistics and zero-order correlations among study 3 variables

	*M* (*SD*)	1	2	3	4
1. Actors’ stress appraisals	1.61 (2.86)	-			
2. Actors’ approach behaviors	5.16 (1.37)	.101^	-		
3. Actors’ avoidance behaviors	2.60 (1.37)	− .095^	.050^	-	
4. Actors’ perceived responsiveness, intimacy, and closeness	5.86 (1.33)	.219	.549	− .245	-
5. Partners’ stress appraisals	1.61 (2.86)	.013^	.173	.013^	.160
6. Partners’ approach behaviors	5.16 (1.37)	.173	.262	− .026^	.351
7. Partners’ avoidance behaviors	2.60 (1.37)	.013^	− .026^	.156	− .196

All correlations are significant at *p* < .01, except for those denoted with ^

#### Stress Appraisals in Anticipation of a Conflict Discussion and Approach and Avoidance Behaviors During the Discussion (Hypothesis 1)

Approach and avoidance behaviors (in separate analyses) were regressed on actors’ and partners’ stress appraisals. The results shown in Table 9 support H1p but not H1a: when partners anticipated the upcoming conversation as more challenging and less threatening, actors engaged in more approach-oriented behaviors. No other effects were significant, though actor effects were trending in the expected direction.

**Table 9:** The associations between actors’ and partners’ anticipatory stress appraisals and approach and avoidance behaviors during the conversation (study 3, hypothesis 1)

	Approach behaviors					Avoidance behaviors				
Predictor variables	*B*	*SE*	*t*	*p*	*r*	*B*	*SE*	*t*	*p*	*r*
Actors’ stress appraisals	0.05	0.03	1.70	.090	.10	− 0.05	0.03	− 1.63	.105	.10
Partners’ stress appraisals	0.08	0.03	2.89	.004	.17	.01	0.03	0.23	.819	.00

#### Associations Between Stress Appraisals (H2) and Behavior (H3) on Feelings of Relationship Security and Well-Being

The results shown in Table 10 provide evidence for hypothesis H2a but not H2p. First, actors (H2a) who appraised the conversation as more challenging and less threatening perceived their partners as more responsive and felt more intimate and closer after the discussion (see left column of Table 10). No significant effects were observed for partners’ stress appraisals (H2p). Both actors’ (H3a) and partners’ (H3p) approach and avoidance behaviors were associated with short-term outcomes. Actors and partners who engaged in more approach and less avoidance behaviors during the conversation perceived their partners as more responsive and felt more intimate and closer with their partners.

**Table 10:** The associations between actors’ and partners’ stress appraisals (hypothesis 2) and approach and avoidance behaviors (hypothesis 3) during the conversation and feelings of relationship security and well-being (study 3, hypothesis 2)

	Responsiveness, intimacy, and closeness					Controlling for relationship satisfaction				
Predictor variables	*B*	*SE*	*t*	*p*	*r*	*B*	*SE*	*t*	*p*	*r*
Actors’ stress appraisals	0.06	0.02	2.62	.009	.16	0.04	0.02	1.75	.082	.11
Actors’ avoidance behaviors	− 0.22	0.04	− 5.30	< .001	.31	− 0.20	0.04	− 4.68	< .001	.28
Actors’ approach behaviors	0.47	0.04	10.88	< .001	.55	0.40	0.04	9.04	< .001	.48
Partners' stress appraisals	0.02	0.02	0.85	.396	.05	0.01	0.02	0.39	.700	.02
Partners’ avoidance behaviors	− 0.14	0.04	− 3.31	.001	.20	− 0.11	0.04	− 2.50	.013	.15
Partners’ approach behaviors	0.20	0.04	4.51	< .001	.26	0.14	0.04	3.19	.002	.19
Actors’ relationship satisfaction	-	-	-	-	-	0.35	0.08	4.31	< .001	.26
Partners’ relationship satisfaction	-	-	-	-	-	0.12	0.08	1.40	.162	.09

#### Ancillary Analyses

Ancillary tests of H1p revealed that the partner effect remained significant after controlling for actors’ and partners’ baseline levels of relationship satisfaction. Ancillary tests of H2a that controlled for baseline levels of relationship satisfaction revealed a similar pattern to the ancillary analyses conducted in studies 1 and 2. The association between actors’ (H2a) stress appraisals and perceived responsiveness and feelings of closeness and intimacy were attenuated (see right column of Table 10). All significant associations between approach- and avoidance-oriented behaviors and feelings of relationship security and well-being remained significant (H3a and H3p). During the review process, we also conducted additional exploratory analyses (see OSM for a summary). The pattern of results did not change when controlling for and examining potential moderation effects of self-report items that assessed actor and partner stress intensity. Moreover, we did not observe interactions between actor’s and partner’s stress appraisals on behavior or feelings of relationship security and well-being.

## Discussion

Findings across three studies highlighted the dyadic nature of stress appraisals and their impact on relationship-relevant behavior and feelings (see Table 11). Results suggested that the way individuals (H1a) and their partners (H1p) appraised stress was associated with behaviors they engaged in during discussions. Appraising stress as more challenging and less threatening was associated with more approach- and less avoidance-oriented relationship behaviors within interactions. However, whose appraisals (actors or partners) were associated with behaviors varied. In study 1, individuals who appraised the upcoming conversation as more challenging and less threatening engaged in more positive-indirect behaviors (closely mirroring our conceptualization of approach-oriented behaviors). In contrast to the actor effects observed in study 1, study 3 revealed that individuals whose partners appraised the upcoming conversation as more challenging and less threatening engaged in more approach-oriented behavior. Finally, results from study 2 revealed the strongest support for predictions, actors’ *and* partners’ stress appraisals were associated with both approach and avoidance behaviors. Together, these results suggest that stress appraisals are uniquely associated with interpersonal behavior within interactions, over and above general relationship satisfaction.

**Table 11:** Summary of results across studies 1, 2, and 3

	Association	Study 1	Study 2	Study 3
(H1a) Actors’ stress appraisals ➔ approach-oriented behavior	+	✓	✓	×
(H1a) Actors’ stress appraisals ➔ avoidance-oriented behavior	−	n/a	✓	×
(H2a) Actors’ stress appraisals ➔ relationship security and well-being	+	✓*	✓*	✓*
(H3a) Actors’ approach behaviors ➔ relationship security and well-being	+	×	✓	✓
(H3a) Actors’ avoidance behaviors ➔ relationship security and well-being	−	n/a	✓	✓
(H1p) Partners’ stress appraisals ➔ approach-oriented behavior	+	×	✓	✓
(H1p) Partners’ stress appraisals ➔ avoidance-oriented behavior	−	n/a	✓	×
(H2p) Partners’ stress appraisals ➔ relationship security and well-being	+	×	✓	×
(H3p) Partners’ approach behaviors ➔ relationship security and well-being	+	×	✓	✓
(H3p) Partners’ avoidance behaviors ➔ relationship security and well-being	−	n/a	×	✓

+  = positive association.—= negative association. ×  = no significant effects observed. ✓ = significant effects observed. * = does not hold when controlling for baseline levels of relationship satisfaction. n/a = not applicable because predictor was not assessed. Stress appraisals were scored such that higher scores indicated greater challenge and less threat

The second set of hypotheses focused on how stress appraisals (H2) and behaviors (H3) were associated with feelings of relationship security and well-being. H2 was partially supported, but with a caveat. Actors’ (studies 1, 2, and 3) and partners’ (study 2) greater challenge and lesser threat appraisals were associated with perceiving partners as more responsive and enhancing feelings of closeness and intimacy. However, baseline levels of actor and partner relationship satisfaction attenuated the actor effects across all studies. In contrast, while partners’ appraisals of stress seemed to provide unique variance in predicting relationship security and well-being (study 2), this pattern did not emerge in studies 1 and 3. We also tested the effects of approach- and avoidance-oriented behaviors on feelings of relationship security and well-being (H3). Although study 1 did not support hypotheses, results across studies 2 and 3 indicated that engaging in more approach-oriented (studies 2 and 3) and less avoidance-oriented behaviors (study 3) was associated with greater feelings of relationship security and well-being, even after controlling for relationship satisfaction.

The current work breaks away from the idea that stress is inherently “bad” or “maladaptive” and instead underscores the importance of examining individual variability in how stress is appraised. That is, the extent to which one appraises stress as more of a challenge and less of a threat is associated with more relationship behaviors that approach incentives and less that avoid threat (Gable & Impett, 2012). Moreover, this research takes an integrative approach to science whereby innovations emerge from bridging different areas rather than carving out new models/theories (e.g., Yeager et al., 2022). Across three studies, intraindividual stress appraisal processes were associated with how individuals behaved toward and perceived one another (Falconier & Kuhn, 2019). This contributes to the growing literature spanning affective and relationship sciences that emphasizes the importance of contextual and relational factors in considering how stress responses occur in dyadic settings (e.g., Bodenmann et al., 2016; Brown et al., 2021; Butler, 2011; English & Eldesouky, 2020; English et al., 2013, 2017; Falconier & Kuhn, 2019; Neff & Karney, 2004; Niven et al., 2009; Waters et al., 2020; West et al., 2017; Zaki & Williams, 2013). Stress appraisal processes may be important in determining whether couples prosper in the face of adversity and whether stress is experienced as adverse or as an opportunity to grow and thrive (e.g., Crum et al., 2020; Feeney & Collins, 2015; Jamieson et al., 2018).

Several limitations should be considered when interpreting this research. First, couples were mostly young, satisfied, White, and heterosexual. Myriad cultural and developmental differences should have notable influences on how individuals appraise and respond to stressors (Falconier et al., 2016; Kayser et al., 2007). Focusing future research endeavors on these sources of heterogeneity will help determine how stress processes function in relationship contexts (Bryan et al., 2021; DiGiovanni et al., 2022). Second, the stress appraisals and behaviors examined here may have notable long-term implications for relationship well-being (Feeney & Collins, 2015; Gable & Gosnell, 2013; Gable & Impett, 2012; Neff & Karney, 2017; Pietromonaco et al., 2022; Reis & Clark, 2013; Rusbult & Van Lange, 2008; Simpson & Rholes, 2017). However, we did not find evidence of these long-term outcomes (see a full report of analyses in the OSM for studies 2 and 3). Third, the correlational nature of the findings and variability of associations across the three studies points to the need for follow-up research using experimental methods to better understand mechanisms. Another interesting area for future work is examining whose appraisals and behaviors matter most in which contexts. For example, we focused on dyadic-interactional conversation paradigms, but other contexts like performative-evaluative contexts (e.g., completing a cooperative task/game, having one person give a speech while the other supports) may moderate stress processes. Fourth, consistent with the BPS model of challenge and threat, we operationalized stress states as a continuum with threat on one end and challenge on the other. Future work may unpack whether resources, demands, and their interaction are differentially and consistently associated with behavior and relationship well-being.^1^

## Conclusion

Individuals’ appraisals of stress are associated with behaviors romantic couples engage in during relationship-relevant conversations. These approach- and avoidance-oriented relationship behaviors were associated with relationship well-being, including responsiveness, closeness, and intimacy. Together, this work places an interpersonal emphasis on the intraindividual stress appraisal processes via a dyadic and close relationships lens.

### Supplementary Information

Below is the link to the electronic supplementary material.

Supplementary file1 (DOCX 122 KB)

## Footnotes

1 We conducted exploratory analyses with resources, demands, and their interaction. See the OSM for a summary of these analyses.

## Additional Information

### Data Availability

The three datasets generated and analyzed for the manuscript are not publicly available due to potential for individual privacy to be compromised but are available from the corresponding author on reasonable request. Studies 1 and 3 were pre-registered. Links are also embedded within the manuscript: 10.17605/OSF.IO/KCAQN; 10.17605/OSF.IO/WRGJH.
