# Supplementary material for: Examining Dyadic Stress Appraisal Processes Within Romantic Relationships from a Challenge and Threat Perspective
Source: Affect Sci. 2024 Jan 30;5(2):69–81. doi: 10.1007/s42761-024-00235-3 (PMC11264650; doi:10.1007/s42761-024-00235-3)
Supplement: Supplementary file 1 — Supplementary file1 (DOCX 122 KB) [file 42761_2024_235_MOESM1_ESM.docx]

Online Supplemental Material (OSM) for:

**Examining dyadic stress appraisal processes within romantic relationships from a challenge and threat perspective**

# Table of Contents

Table of Contents 2

Supplemental Methods 3

Participants 3

Study 1 4

Study 2 6

Measures of Key Constructs 7

Supplemental Results 14

Supplemental Discussion 37

Longitudinal Findings 37

# Supplemental Methods

## Participants

| Table S1 | | | | | | |
| --- | --- | --- | --- | --- | --- | --- |
| *Sample Characteristics for Studies 1, 2, and 3.* | | | | | | |
|  | **Study 1** | | **Study 2** | | **Study 3** | |
|  | ***M (SD)*** | **Range** | ***M (SD)*** | **Range** | ***M (SD)*** | **Range** |
| ***Age*** | 20.21 (2.50) | 18-36 | 20.20 (1.87) | 17-36 | 25.73 (7.10) | 18-78 |
| ***Relationship Length*** | 22.43 (22.49) | 1-124 | 15.57 (11.99) | 2-57 | 41.52 (51.24) | 12-627 |
|  | ***N*** | **%** | ***N*** | **%** | ***N*** | **%** |
| ***Gender/Sex*** |  |  |  |  |  |  |
| Man/Male | 131 | 48.30 | 171 | 48.30 | 142 | 49.70 |
| Woman/Female | 138 | 50.90 | 183 | 51.70 | 144 | 50.30 |
| Other | 2 | 0.70 | 0 | 0 | 0 | 0 |
| ***Race*** |  |  |  |  |  |  |
| White | 235 | 86.70 | 192 | 54.20 |  |  |
| Black/African American | 16 | 5.90 | 26 | 7.30 |  |  |
| Asian | 7 | 2.60 | 93 | 26.30 |  |  |
| Mixed/Other | 13 | 4.80 | 31 | 8.80 |  |  |
| ***Ethnicity*** |  |  |  |  |  |  |
| Hispanic/Latino | 7 | 2.50 | 26 | 7.30 |  |  |
| Non-Hispanic/Latino | 264 | 97.40 | 315 | 89.00 |  |  |
| New Zealand/ Pākehā |  |  |  |  | 110 | 38.5 |
| Māori |  |  |  |  | 1 | 0.3 |
| Pacific Nations |  |  |  |  | 6 | 2.1 |
| Asian |  |  |  |  | 71 | 24.8 |
| Indian |  |  |  |  | 10 | 3.5 |
| European (non-NZ) |  |  |  |  | 30 | 10.5 |
| Mixed/Other |  |  |  |  | 58 | 27.2 |
| *Note.* Age is measured in years. Relationship length is measured in months. Note, percentages from some categories do not add up to 100% due to missing or excluded data. | | | | | | |

## Study 1

**Extra-dyadic Problem Discussion Paradigm & Co-Rumination Manipulation**

During the lab session, experimenters gave participants the following instructions based on experimental condition assignment.

For participants assigned to the natural condition:

*“In a moment, this wall divider is going to be removed and you and your partner will engage in a conversation where one of you will be assigned to disclose one of the personal problems you wrote about previously.”*

[Read to DISCLOSERS] *“You have been assigned to disclose your problem regarding _________ to your partner. You may start the conversation off by describing the problem to your partner, giving them enough detail to get into the conversation. Then talk about the problem as you naturally would.”*

[Read to RESPONDERS] *“Your partner has been assigned to disclose a problem to you regarding ________. They will likely start off by describing the problem to you. After that, talk about the problem as you naturally would.”*

*“Do you have any questions about the procedure or topic?”*

*“If you need a reminder of the instructions, you can refer to this card*.” [Give participant instruction card]

*“Please take a few moments to gather your thoughts related to this topic. Try to stay still during this time as we will be taking physio signals. When we tell you over the intercom, answer the questions on the computer screen.”*

For participants assigned to the co-rumination condition:

*“In a moment, this wall divider is going to be removed and you and your partner will engage in a conversation where one of you will be assigned to disclose one of the personal problems you wrote about previously.”*

*“Throughout the conversation, we ask that you and your partner do the following:”*

*“First, stay on topic. If you or your partner begin discussing something other than the problem at hand, bring each other back on topic.”*

*“Second, really try to figure out the causes and consequences of the problem. Be sure that you or your partner discuss the potential origins of the problem and the future consequences that may result from it.”*

*“Third, go over the problem again. Start from the beginning and try to think about the problem from a different perspective. Consider an aspect of the problem that has yet to be discussed in order to think more deeply about the situation.”*

*“Fourth, uncover and really dig into negative emotions. Throughout the conversation, focus on the negative emotions each of you are feeling or could imagine feeling and why you might feel that way. Really push each other to understand the negative emotions involved in the situation. Some examples of negative emotions include: irritated, sad, anxious, depressed, scared, distressed, shameful, or frustrated.”*

*“Remember, your goal for the conversation isn’t to come to a solution to the problem, but to: stay on topic, try to figure out the causes and consequences of the problem, go over the problem again, and uncover and dig into negative emotions. Be sure to follow these directions, but in a way that feels natural to you and how you would normally discuss these types of issues with your partner.”*

[Read to DISCLOSERS] “*You have been assigned to disclose your problem regarding _________ to your partner. You may start the conversation off by describing the problem to your partner, giving your partner enough detail to start the conversation. Then talk about the problem as you naturally would.”*

[Read to RESPONDERS] “*Your partner has been assigned to disclose a problem to you regarding ________. After they describe the problem, engage with them as you naturally would.”*

*“Do you have any questions about the procedure or topic?”*

*“If you need a reminder of the instructions, you can refer to this card*.” [Give participant instruction card]

*“Please take a few moments to gather your thoughts related to this topic. Try to stay still during this time as we will be taking physio signals. When we tell you over the intercom, answer the questions on the computer screen.”*

At this time, experimenters left the participant room to allow participants to gather their thoughts and complete a brief questionnaire. Then, experimenters returned to the participant room, repeated the conversation instructions, and removed the wall dividing the participants.

Participants were instructed not to begin their conversation until instructed over the loudspeaker. The experimenters then left the room. The operator instructed the participants to begin their conversation. Participants discussed the problem for 8 minutes. Following the conversation, the wall was replaced, and participants rested while participants completed additional questionnaires.

## Study 2

**Conversation Paradigm & Discussion Manipulation**

Couples were randomly assigned to receive instructions for one of two conversations.

In one of the conversations, couples engaged in an 8-minute discussion about the things they disliked most about each other. One member of the dyad was specifically instructed to reveal their dislikes first and discuss them with their partner:

*“You are now going to engage in another discussion with your romantic partner. People are not perfect; everyone has flaws. Just like there are things we don’t like about ourselves, there are often habits, attitudes, or behaviors we don’t like about our romantic partner and may even cause conflict or problems in the relationship. For the next conversation, you and your significant other will engage in an 8-minute discussion about the top 3 things that annoy you the most about your partner or consider to be ‘pet-peeves.’ For each annoyance or pet-peeve, explain to your partner, in detail, why you picked it. Like during the last conversation you will have 2 minutes to explain your choices to your partner, and 2 minutes to discuss. We will notify you when the 4-minutes are up and then the other person will have their turn.”*

In the other conversation, couples engaged in an 8-minute conversation about the top three ways in which they depend on their partners. As with the other condition, one member of the dyad was specifically instructed to reveal their top three choices first:

“*You are now going to engage in another discussion with your romantic partner. Romantic partners can be a primary source of support, closeness, and intimacy. During times of stress, we often rely on our partners to be there for us. Over time, our romantic partners can become the people we are the closest and most intimate with. For the next conversation, you and your significant other will engage in an 8-minute discussion about the top 3 ways you depend on your partner. For each way you depend, explain to your partner, in detail, why you picked it. Like during the last conversation you will have 2 minutes to explain your choices to your partner, and 2 minutes to discuss. We will notify you when the 4-minutes are up and then the other person will have their turn.”*

After receiving interaction instructions, participants remained in their private testing rooms for a preparation period during which they were given three minutes to “gather their thoughts” and prepare for the conversation.

## Measures of Key Constructs

### Self-reported stress appraisals

#### Study 1

DIRECTIONS: For the following items, please rate how true each statement is regarding the upcoming conversation.

1 = Strongly disagree, 2 = Disagree, 3 = Somewhat disagree, 4 = Neutral, 5 = Somewhat agree, 6 = Agree, 7 = Strongly agree

- Demands
  - The upcoming conversation is very demanding
  - The upcoming conversation will take a lot of effort to complete
- Resources
  - I have the abilities to do well during the conversation
  - I'm the kind of person that does well with these types of conversations

#### Study 2

DIRECTIONS: Please select a number after each statement to indicate how you are feeling right now regarding the conversation you are about to have

1 = Strongly Disagree, 4 = Neutral, 7 = Strongly Agree

- Demands
  - The upcoming conversation is very demanding
  - The upcoming conversation will take a lot of effort to complete
- Resources
  - I have the abilities to do well during the conversation
  - I’m the kind of person that does well with these types of conversations

#### Study 3

DIRECTIONS: Thinking about the issue, to what extent do YOU agree with the following?

1 = not at all, 7 = very much

- Demands
  - There is little I can do to solve this issue
  - I have little control over this issue
- Resources
  - I am able to do the things needed to settle this issue
  - I have the capability to solve this issue

### Self-Reported Approach/Avoidance-Oriented Behaviors Questionnaire

#### Study 1

*N/A*

#### Study 2

DIRECTIONS: Please rate the following items on a scale of 1 to 7 to indicate how much agree or disagree that each statement reflects how you behaved during the previous conversation

1 = Strongly Disagree, 4 = Neutral, 7 = Strongly Agree

- Approach-oriented behaviors
  - I made sure my partner knows that I love him/her
  - I reassured my partner that everything was going to be okay
  - I acknowledged my partner's concerns and fears
  - I tried to get my partner to open up during the conversation
  - I tried to generate back-and -forth discussion to get my partner talking and engaged
  - I acknowledged the good things my partner had done for the relationship
- Avoidance-oriented behaviors
  - I avoided being too direct or blunt with my partner
  - I avoided saying anything that could hurt my partner's feelings
  - I hid my negative thoughts or feelings about my partner
  - I exaggerated how positively I thought or felt about my partner
  - I downplayed the severity of the problem

#### Study 3

DIRECTIONS: The following series of questions relate to how YOU experienced the discussion.

Please rate the following questions according to how YOU felt, thought and behaved during the discussion.

1 = Not at all, 7 = Very much, * = new item (relative to Study 2)

- Approach-oriented behaviors
  - I made sure my partner knows that I love him/her
  - I reassured my partner that everything was going to be okay
  - I tried to get my partner to open up during the conversation
  - I tried hard to get my partner talking and engaged
  - I let my partner know I support him/her*
  - I let my partner know I was there for him/her*
  - I tried to ensure my partner was alright*
- Avoidance-oriented behaviors
  - I avoided being too direct or blunt with my partner
  - I avoided saying anything that could hurt my partner's feelings
  - I hid my negative thoughts or feelings about my partner
  - I exaggerated how positively I thought or felt about my partner
  - I avoided disagreeing with my partner*
  - I withheld potentially upsetting information from my partner*
  - I tried to indirectly raise issues to minimize conflict*

### Feelings of Relationship Security and Well-Being

#### Study 1

***Responsiveness, Intimacy, and Closeness***

DIRECTIONS: To what extent do you agree or disagree that you feel...

1 = Strongly disagree, 2 = Disagree, 3 = Somewhat disagree, 4 = Neither agree nor disagree, 5 = Somewhat agree, 6 = Agree, 7 = Strongly agree

- cared for/loved by your partner?
- close/intimate with your partner?
- comforted/reassured by your partner?
- understood/validated by your partner?
- supported/helped by your partner?
- accepted/valued by your partner?
- warm/affectionate towards your partner?

#### Study 2

***Perceived Partner Responsiveness***

DIRECTIONS: During the conversation with my partner that I just had, I got the feeling that my romantic partner:

1 = Not at all True, 3 = Somewhat True, 5 = Very True, 7 = Completely True

- saw the “real” me
- “got the facts right” about me
- esteemed me, shortcomings and all
- knew me well
- valued and respected the whole package that is the “real” me
- understood me
- really listened to me
- expressed liking and encouragement for me
- seemed interested in what I am thinking and feeling
- valued my abilities and opinions
- was on “the same wavelength” with me
- was responsive to my needs

***Intimacy and Closeness***

DIRECTIONS: Thinking about the topic, to what extent:

1 = not at all, 7 = very much

- Do you feel closer with your partner
- Do you feel more intimate with your partner

#### Study 3

***Responsiveness, Intimacy, and Closeness***

All items were completed using a 7-point Likert scale (1 = not at all, 7 = very much). Items were presented and in reference to the conflict discussion that had just occurred.

- I felt …understood/validated by my partner
- I felt …accepted/valued by my partner
- I felt …cared for/loved by my partner
- I felt …comforted/reassured by my partner
- I felt …close/intimate with my partner
- I felt …warm/affectionate towards my partner

### Baseline Assessments of Relationship Satisfaction

#### Study 1

Please indicate the degree of happiness, all things considered, of your relationship

0 = extremely unhappy

1 = fairly unhappy

2 = a little unhappy

3 = happy

4 = very happy

5 = extremely happy

6 = perfect

In general, how often do you think that things between you and your partner are going well?

0 = never

1 = rarely

2 occasionally

3 = more often than not

4 = most of the time

5 = all of the time

[The following items were answered on this scale: (0 = not at all true, 1 = a little true, 2 = somewhat true, 3 = mostly true, 4 = almost completely true, 5 = completely true).]

- Our relationship is strong
- My relationship with my partner makes me happy
- I have a warm and comfortable relationship with my partner
- I really feel like part of a team with my partner

[The following items were answered on this scale: (0 = not at all, 1 = a little, 2 = somewhat, 3 = mostly, 4 = almost completely, 5 = completely).]

- How rewarding is your relationship with your partner?
- How well does your partner meet your needs?
- To what extent has your relationship met your original expectations?
- In general, how satisfied are you with your relationship?

For each of the following items, select the answer that best describes how you feel about your relationship. Base your responses on your first impressions and immediate feelings about the item.

0 = boring, 5 = interesting

0 = bad, 5 = good

0 = empty, 5 = full

0 = fragile, 5 = sturdy

0 = discouraging, 5 = hopeful

0 = enjoyable, 5 = miserable [reversed]

#### Study 2

Please indicate the degree of happiness, all things considered, of your relationship

0 = extremely unhappy

1 = fairly unhappy

2 = a little unhappy

3 = happy

4 = very happy

5 = extremely happy

6 = perfect

In general, how often do you think that things between you and your partner are going well?

0 = never

1 = rarely

2 occasionally

3 = more often than not

4 = most of the time

5 = all of the time

[The following items were answered on this scale: (0 = not at all true, 1 = a little true, 2 = somewhat true, 3 = mostly true, 4 = almost completely true, 5 = completely true).]

- Our relationship is strong
- My relationship with my partner makes me happy
- I have a warm and comfortable relationship with my partner
- I really feel like part of a team with my partner

[The following items were answered on this scale: (0 = not at all, 1 = a little, 2 = somewhat, 3 = mostly, 4 = almost completely, 5 = completely).]

- How rewarding is your relationship with your partner?
- How well does your partner meet your needs?
- To what extent has your relationship met your original expectations?
- In general, how satisfied are you with your relationship?

For each of the following items, select the answer that best describes how you feel about your relationship. Base your responses on your first impressions and immediate feelings about the item.

0 = boring, 5 = interesting

0 = bad, 5 = good

0 = empty, 5 = full

0 = fragile, 5 = sturdy

0 = discouraging, 5 = hopeful

0 = enjoyable, 5 = miserable [reversed]

#### Study 3

All items were completed using a 7-point Likert scale.

- I feel satisfied with our relationship.
- Our relationship is much better than others’ relationships.
- Our relationship is close to ideal.
- Our relationship makes me very happy.
- Our relationship does a good job of fulfilling my needs for intimacy, companionship, etc.

### Communication Strategies Coding Scheme

Adapted from N.C. Overall by A.M. Gresham and B.J. Peters, 2020

Positive Indirect (PI) behaviors are passive responses to the topic, including attempting to soften the impact of problems, being loyal to the partner, giving the partner the benefit of the doubt (e.g., attempts to inhibit hurt reactions to the partner’s negativity), minimizing or downplaying the severity of the topic. The focus is on maintaining positivity and not hurting the partner, and less on expressing the problems and searching for solutions. The ratings should represent the degree to which the individual displayed **positive indirect loyalty behaviors** (e.g., acknowledging the topic’s importance but without strong effort to change, softening conflict by minimizing the topic, validating the partner and focusing on positive aspects of the partner/relationship, showing optimism for improvement but providing few or no suggestions, trying not to react negatively to the topic or partner negativity). Behaviors that fit into this category include:

- Softening impact by minimizing the topic (“it’s not that big of a deal”, “you got this”, “we will make it work”), recognizing the partner’s efforts to find a solution or pointing out other good characteristics of the partner, and validating the partner’s point of view (even when they don’t agree with that point of view)
- Attempts to minimize issues or conflict by using charm, affection, and positive affect (e.g., humor)
- Showing loyalty by attempting not to react negatively to the topic or partner’s negative behavior within the interaction
- Acknowledging the importance of the topic but not actively working toward a solution, exhibiting optimism by believing and hoping it will improve

***Protocol***

- You will watch each 8-minute conversation in 4-minute segments. The conversation starts when you hear “*you may begin*” and ends 8 minutes after. If the conversation exceeds 8 minutes, stop coding after 8 minutes are over. If the conversation goes under 8 minutes, treat the end of the conversation as the 8-minute mark (i.e., don’t code 1’s for everything just because the conversation ended- just stop coding when the conversation ends).
- For each segment:
- **Rate** the degree to which the individual displays behaviors using the scale below.
- After viewing an agreed upon number of interactions, touch base with your partner. Review all scores to make sure they match.
  - If a score doesn’t match, discuss with your partner. Decide on which score to make the final. Record that in the AGREEMENT tab. But DON’T change your original score in your tab- keep those the same.

# Supplemental Results

## Ancillary Analyses

*Study 1*

Analyses including experimental condition, conversation role, and all possible interactions did not alter the main findings. See Table S2 for tests of Hypothesis 1 and Table S3 for Hypotheses 2 and 3. Disclosers engaged in less positive-indirect behaviors during the conversation than responders (see Table S2). Additionally, a significant Partner Positive-Indirect behavior by Role interaction suggested that for disclosers who had partners who engaged in more positive-indirect behaviors they perceived their partner as more responsive and felt closer and more intimate (*B* = .17, *SE* = .07, *t* = 2.31, *p* = .023, *r* = .21) whereas the association between partners’ indirect-behavior and closeness was attenuated for responders (*B* = -.09, *SE* = .09, *t* = -1.03, *p* = .304, *r* = .10).

Ancillary analyses also included controlling for actors’ and partners’ relationship satisfaction are shown in Tables S4 through S5. As noted in the main manuscript, all significant effects reported in the main pre-registered analyses for hypothesis 1 remained significant. However, additional analyses for hypothesis 2a revealed that actors’ greater relationship satisfaction was significantly associated with greater closeness and intimacy after the conversation, and this strong association weakened the main effects of actors’ stress appraisals on closeness and intimacy.

| **Table S2** | | | | | |
| --- | --- | --- | --- | --- | --- |
| *The associations between actors’ (H1a) and partners’ (H1p) anticipatory stress appraisals and positive-indirect behaviors during the conversation controlling for experimental condition and role in Study 1*. | | | | | |
|  | *B* | *SE* | *t* | *p* | *r* |
| Actors' Stress Appraisals | 0.07 | 0.02 | 3.31 | 0.001 | 0.22 |
| Partners' Stress Appraisals | -0.01 | 0.02 | -0.67 | 0.507 | 0.04 |
| Condition | -0.05 | 0.04 | -1.20 | 0.231 | 0.11 |
| Role | -0.16 | 0.04 | -4.10 | <.001 | 0.35 |
| Condition × Role | 0.08 | 0.04 | 2.13 | 0.036 | 0.19 |
| Actors' Stress Appraisals × Condition | -0.02 | 0.02 | -1.21 | 0.228 | 0.08 |
| Actors' Stress Appraisals × Role | 0.00 | 0.02 | -0.04 | 0.966 | 0.00 |
| Actors' Stress Appraisals × Condition × Role | 0.02 | 0.02 | 0.91 | 0.363 | 0.06 |
| Partners' Stress Appraisals × Condition | 0.02 | 0.02 | 0.87 | 0.387 | 0.06 |
| Partners' Stress Appraisals × Role | 0.03 | 0.02 | 1.36 | 0.176 | 0.10 |
| Partners' Stress Appraisals × Condition × Role | -0.03 | 0.02 | -1.71 | 0.090 | 0.12 |

| **Table S3** | | | | | |
| --- | --- | --- | --- | --- | --- |
| *The associations between actors’ (H2a) and partners’ (H2p) stress appraisals and positive-indirect behaviors (H3a, H3p) on responsiveness, closeness, and intimacy controlling for experimental condition and role in Study 1*. | | | | | |
|  | *B* | *SE* | *t* | *p* | *r* |
| Actors' Stress Appraisals | 0.05 | 0.02 | 2.80 | 0.006 | 0.19 |
| Actors' Positive-Indirect Behaviors | 0.01 | 0.06 | 0.19 | 0.853 | 0.01 |
| Partners' Stress Appraisals | 0.02 | 0.02 | 1.14 | 0.255 | 0.08 |
| Partners' Positive-Indirect Behaviors | 0.04 | 0.06 | 0.68 | 0.496 | 0.05 |
| Condition | 0.07 | 0.04 | 1.67 | 0.097 | 0.16 |
| Role | 0.01 | 0.03 | 0.16 | 0.872 | 0.02 |
| Condition × Role | 0.02 | 0.03 | 0.53 | 0.594 | 0.05 |
| Actors' Stress Appraisals × Condition | 0.01 | 0.02 | 0.48 | 0.630 | 0.03 |
| Actors' Stress Appraisals × Role | 0.02 | 0.02 | 0.82 | 0.414 | 0.06 |
| Actors' Stress Appraisals × Condition × Role | 0.00 | 0.02 | -0.23 | 0.822 | 0.02 |
| Partners' Stress Appraisals × Condition | 0.00 | 0.02 | -0.28 | 0.783 | 0.02 |
| Partners' Stress Appraisals × Role | -0.01 | 0.02 | -0.68 | 0.496 | 0.05 |
| Partners' Stress Appraisals × Condition × Role | 0.02 | 0.02 | 0.91 | 0.363 | 0.07 |
| Actors' Positive-Indirect Behaviors × Condition | -0.02 | 0.06 | -0.38 | 0.702 | 0.03 |
| Actors' Positive-Indirect Behaviors × Role | -0.12 | 0.06 | -1.90 | 0.059 | 0.14 |
| Actors' Positive-Indirect Behaviors × Condition × Role | 0.03 | 0.06 | 0.53 | 0.598 | 0.04 |
| Partners' Positive-Indirect Behaviors × Condition | -0.01 | 0.06 | -0.25 | 0.801 | 0.02 |
| Partners' Positive-Indirect Behaviors × Role | 0.13 | 0.06 | 2.21 | 0.028 | 0.16 |
| Partners' Positive-Indirect Behaviors × Condition × Role | -0.01 | 0.06 | -0.08 | 0.933 | 0.01 |

***Figure S1.*** *The associations between partners’ positive-indirect behaviors and actors’ feelings of responsiveness, closeness, and intimacy after the conversation as a function of conversation role (Study 1: ancillary test of H3p).* *Note, minimum observed value of -.48 was used instead of -1 SD because -1 SD went outside the observed values of positive-indirect behaviors.*

| **Table S4** | | | | | |
| --- | --- | --- | --- | --- | --- |
| *The associations between actors’ (H1a) and partners’ (H1p) anticipatory stress appraisals and positive-indirect behaviors during the conversation controlling for actors’ and partners’ relationship satisfaction in Study 1*. | | | | | |
|  | *B* | *SE* | *t* | *p* | *r* |
| Actors' Stress Appraisals | 0.06 | 0.02 | 2.88 | 0.004 | 0.20 |
| Partners' Stress Appraisals | 0.01 | 0.02 | 0.33 | 0.739 | 0.02 |
| Actors' Relationship Satisfaction | 0.00 | 0.01 | 0.62 | 0.539 | 0.05 |
| Partners' Relationship Satisfaction | 0.00 | 0.01 | -0.17 | 0.864 | 0.01 |

| **Table S5** | | | | | |
| --- | --- | --- | --- | --- | --- |
| *The associations between actors’ (H2a) and partners’ (H2p) stress appraisals and positive-indirect behaviors (H3a, H3p) on responsiveness, closeness, and intimacy controlling for actors’ relationship satisfaction in Study 1*. | | | | | |
|  | *B* | *SE* | *t* | *p* | *r* |
| Actors' Stress Appraisals | 0.02 | 0.01 | 1.42 | 0.158 | 0.10 |
| Actors' Positive-Indirect Behaviors | 0.02 | 0.04 | 0.46 | 0.650 | 0.03 |
| Partners' Stress Appraisals | 0.00 | 0.01 | -0.15 | 0.883 | 0.01 |
| Partners' Positive-Indirect Behaviors | 0.04 | 0.04 | 0.96 | 0.337 | 0.06 |
| Actors' Relationship Satisfaction | 0.05 | 0.00 | 16.57 | 0.000 | 0.79 |
| *Note*. Analyses including partners’ relationship satisfaction did not converge and was removed from the analysis. | | | | | |

*Study 2*

*Experimental Condition and Conversation Role.* All significant effects reported in the main manuscript remained when fully crossing condition and role except for partners’ stress appraisals predicting avoidance-oriented relationship behaviors (H1p). There was one significant actor stress appraisal × condition effect (see Table S7 and Figure S2), suggesting that the association between greater challenge (less threat) appraisals and greater avoidance-oriented behaviors was significant in the dependence condition (*B* = -.08, *SE* = .04, *t* = -4.79, *p <* .001, *r* = .27) but not the pet-peeve condition (*B* = -.04, *SE* = .03, *t* = -1.024, *p* = .306, *r* = .06).

*Relationship satisfaction*. When including actors’ and partners’ relationship satisfaction in models for tests of hypothesis 1, three of the four results remained significant, however, the association between partners’ greater challenge and less threat appraisals and actors’ less avoidance behaviors was no longer significant. Tests of hypothesis 2 revealed that the direct effects of actors’ stress appraisals on feelings of responsiveness, intimacy, and closeness were attenuated. All other significant effects remained.

| **Table S6** | | | | | |
| --- | --- | --- | --- | --- | --- |
| *The associations between actors’ and partners’ anticipatory stress appraisals and approach-oriented behaviors during the conversation (Hypothesis 1) controlling for experimental condition and role in Study 2*. | | | | | |
|  | *B* | *SE* | *t* | *p* | *r* |
| Actors' Stress Appraisals | 0.08 | 0.02 | 4.02 | 0.000 | 0.22 |
| Partners' Stress Appraisals | 0.05 | 0.02 | 2.37 | 0.018 | 0.13 |
| Condition | 0.01 | 0.06 | 0.12 | 0.904 | 0.01 |
| Role | 0.04 | 0.05 | 0.74 | 0.463 | 0.06 |
| Condition × Role | 0.06 | 0.05 | 1.03 | 0.303 | 0.08 |
| Actors' Stress Appraisals × Condition | 0.03 | 0.02 | 1.31 | 0.193 | 0.07 |
| Actors' Stress Appraisals × Role | 0.00 | 0.02 | -0.19 | 0.849 | 0.01 |
| Actors' Stress Appraisals × Condition × Role | -0.03 | 0.02 | -1.42 | 0.156 | 0.08 |
| Partners' Stress Appraisals × Condition | 0.00 | 0.02 | -0.23 | 0.821 | 0.01 |
| Partners' Stress Appraisals × Role | -0.01 | 0.02 | -0.67 | 0.504 | 0.04 |
| Partners' Stress Appraisals × Condition × Role | 0.01 | 0.02 | 0.53 | 0.599 | 0.03 |

| **Table S7** | | | | | |
| --- | --- | --- | --- | --- | --- |
| *The associations between actors’ and partners’ anticipatory stress appraisals and avoidance-oriented behaviors during the conversation (Hypothesis 1) controlling for experimental condition and role in Study 2*. | | | | | |
|  | *B* | *SE* | *t* | *p* | *r* |
| Actors' Stress Appraisals | -0.11 | 0.03 | -4.19 | <0.001 | 0.23 |
| Partners' Stress Appraisals | -0.05 | 0.03 | -1.88 | 0.061 | 0.11 |
| Condition | -0.07 | 0.08 | -0.87 | 0.385 | 0.07 |
| Role | 0.10 | 0.06 | 1.64 | 0.103 | 0.13 |
| Condition × Role | 0.05 | 0.06 | 0.87 | 0.386 | 0.07 |
| Actors' Stress Appraisals × Condition | -0.07 | 0.03 | -2.79 | 0.006 | 0.16 |
| Actors' Stress Appraisals × Role | 0.00 | 0.03 | -0.05 | 0.963 | 0.00 |
| Actors' Stress Appraisals × Condition × Role | 0.01 | 0.03 | 0.28 | 0.780 | 0.02 |
| Partners' Stress Appraisals × Condition | -0.03 | 0.03 | -1.37 | 0.173 | 0.08 |
| Partners' Stress Appraisals × Role | 0.01 | 0.03 | 0.54 | 0.590 | 0.03 |
| Partners' Stress Appraisals × Condition × Role | -0.02 | 0.03 | -0.82 | 0.414 | 0.05 |

***Figure S2.*** *The associations between actors’ stress appraisals and actors’ avoidance-oriented behaviors during the conversation as a function of study condition (Study 2: ancillary test of H1a).*

| **Table S8** | | | | | |
| --- | --- | --- | --- | --- | --- |
| *The associations between actors’ and partners’ anticipatory stress appraisals and approach-oriented behaviors during the conversation (Hypothesis 1) controlling for actors’ and partners’ relationship satisfaction in Study 2*. | | | | | |
|  | *B* | *SE* | *t* | *p* | *r* |
| Actors' Stress Appraisals | 0.04 | 0.02 | 2.10 | 0.037 | 0.12 |
| Partners' Stress Appraisals | 0.05 | 0.02 | 2.57 | 0.011 | 0.14 |
| Actors' Relationship Satisfaction | 0.03 | 0.00 | 6.97 | 0.000 | 0.39 |
| Partners' Relationship Satisfaction | -0.01 | 0.00 | -1.88 | 0.061 | 0.11 |

| **Table S9** | | | | | |
| --- | --- | --- | --- | --- | --- |
| *The associations between actors’ and partners’ anticipatory stress appraisals and avoidance-oriented behaviors during the conversation (Hypothesis 1) controlling for actors’ and partners’ relationship satisfaction in Study 2*. | | | | | |
|  | *B* | *SE* | *t* | *p* | *r* |
| Actors' Stress Appraisals | -0.07 | 0.03 | -2.76 | 0.006 | 0.15 |
| Partners' Stress Appraisals | -0.04 | 0.03 | -1.49 | 0.138 | 0.08 |
| Actors' Relationship Satisfaction | -0.03 | 0.01 | -4.38 | 0.000 | 0.25 |
| Partners' Relationship Satisfaction | 0.00 | 0.01 | -0.51 | 0.613 | 0.03 |

| **Table S10** | | | | | |
| --- | --- | --- | --- | --- | --- |
| *The associations between actors’ and partners’ stress appraisals and approach- and avoidance- oriented behaviors on feelings of responsiveness, intimacy, and closeness (Hypotheses 2 and 3) controlling for experimental condition and role in Study 2*. | | | | | |
|  | *B* | *SE* | *t* | *p* | *r* |
| Actors' Stress Appraisals | 0.04 | 0.02 | 2.43 | 0.016 | 0.14 |
| Actors' Avoidance-Oriented Behaviors | -0.17 | 0.03 | -5.06 | <0.001 | 0.29 |
| Actors' Approach-Oriented Behaviors | 0.33 | 0.04 | 7.25 | <0.001 | 0.39 |
| Partners' Stress Appraisals | 0.05 | 0.02 | 2.99 | 0.003 | 0.17 |
| Partners' Avoidance-Oriented Behaviors | 0.02 | 0.03 | 0.69 | 0.490 | 0.04 |
| Partners' Approach-Oriented Behaviors | 0.09 | 0.04 | 2.03 | 0.043 | 0.12 |
| Condition | 0.04 | 0.04 | 0.83 | 0.408 | 0.07 |
| Role | -0.01 | 0.04 | -0.18 | 0.861 | 0.01 |
| Condition × Role | 0.02 | 0.04 | 0.44 | 0.660 | 0.04 |
| Actors' Stress Appraisals × Condition | 0.01 | 0.02 | 0.37 | 0.712 | 0.02 |
| Actors' Stress Appraisals × Role | 0.02 | 0.02 | 1.21 | 0.226 | 0.07 |
| Actors' Stress Appraisals × Condition × Role | 0.01 | 0.02 | 0.78 | 0.439 | 0.05 |
| Actors' Avoidance-Oriented Behaviors × Condition | 0.01 | 0.03 | 0.27 | 0.790 | 0.02 |
| Actors' Avoidance-Oriented Behaviors × Role | 0.00 | 0.04 | -0.09 | 0.928 | 0.01 |
| Actors' Avoidance-Oriented Behaviors × Condition × Role | 0.07 | 0.04 | 1.93 | 0.055 | 0.12 |
| Actors' Approach-Oriented Behaviors × Condition | -0.01 | 0.04 | -0.16 | 0.877 | 0.01 |
| Actors' Approach-Oriented Behaviors × Role | -0.02 | 0.05 | -0.42 | 0.675 | 0.02 |
| Actors' Approach-Oriented Behaviors × Condition × Role | -0.03 | 0.05 | -0.62 | 0.537 | 0.04 |
| Partners' Stress Appraisals × Condition | 0.00 | 0.02 | -0.25 | 0.805 | 0.01 |
| Partners' Stress Appraisals × Role | 0.01 | 0.02 | 0.69 | 0.493 | 0.04 |
| Partners' Stress Appraisals × Condition × Role | 0.01 | 0.02 | 0.51 | 0.608 | 0.03 |
| Partners' Avoidance-Oriented Behaviors × Condition | 0.01 | 0.03 | 0.36 | 0.723 | 0.02 |
| Partners' Avoidance-Oriented Behaviors × Role | 0.04 | 0.04 | 1.05 | 0.295 | 0.06 |
| Partners' Avoidance-Oriented Behaviors × Condition × Role | -0.03 | 0.04 | -0.87 | 0.385 | 0.05 |
| Partners' Approach-Oriented Behaviors × Condition | -0.06 | 0.04 | -1.43 | 0.154 | 0.08 |
| Partners' Approach-Oriented Behaviors × Role | 0.08 | 0.05 | 1.72 | 0.087 | 0.10 |
| Partners' Approach-Oriented Behaviors × Condition × Role | 0.00 | 0.05 | -0.01 | 0.990 | 0.00 |

| **Table S11** | | | | | |
| --- | --- | --- | --- | --- | --- |
| *The associations between actors’ and partners’ stress appraisals and approach- and avoidance- oriented behaviors on feelings of responsiveness, intimacy, and closeness (Hypotheses 2 and 3) controlling for actors’ and partners’ relationship satisfaction in Study 2*. | | | | | |
|  | *B* | *SE* | *t* | *p* | *r* |
| Actors' Stress Appraisals | 0.02 | 0.01 | 1.58 | 0.116 | 0.09 |
| Actors' Avoidance-Oriented Behaviors | -0.10 | 0.03 | -3.24 | 0.001 | 0.18 |
| Actors' Approach-Oriented Behaviors | 0.24 | 0.04 | 5.68 | <0.001 | 0.31 |
| Partners' Stress Appraisals | 0.05 | 0.01 | 3.60 | <0.001 | 0.20 |
| Partners' Avoidance-Oriented Behaviors | 0.03 | 0.03 | 0.87 | 0.383 | 0.05 |
| Partners' Approach-Oriented Behaviors | 0.09 | 0.04 | 2.23 | 0.027 | 0.13 |
| Actors' Relationship Satisfaction | 0.03 | 0.00 | 6.85 | 0.000 | 0.38 |
| Partners' Relationship Satisfaction | 0.00 | 0.00 | -0.74 | 0.461 | 0.04 |

***Study 3***

As reported in the main manuscript, ancillary tests including actors’ and partners’ baseline relationship satisfaction did not alter the observed partner effect between stress appraisals and approach-oriented behaviors.

| **Table S12** | | | | | |
| --- | --- | --- | --- | --- | --- |
| *The associations between actors’ and partners’ anticipatory stress appraisals and approach-oriented behaviors during the conversation (Hypothesis 1) controlling for actors’ and partners’ relationship satisfaction in Study 3*. | | | | | |
|  | *B* | *SE* | *t* | *p* | *r* |
| Actors' Stress Appraisals | 0.01 | 0.03 | 0.30 | 0.767 | 0.02 |
| Partners' Stress Appraisals | 0.06 | 0.03 | 2.04 | 0.043 | 0.12 |
| Actors' Relationship Satisfaction | 0.50 | 0.11 | 4.60 | <.001 | 0.28 |
| Partners' Relationship Satisfaction | 0.11 | 0.11 | 1.03 | 0.305 | 0.07 |

| **Table S13** | | | | | |
| --- | --- | --- | --- | --- | --- |
| *The associations between actors’ and partners’ anticipatory stress appraisals and avoidance-oriented behaviors during the conversation (Hypothesis 1) controlling for actors’ and partners’ relationship satisfaction in Study 3*. | | | | | |
|  | *B* | *SE* | *t* | *p* | *r* |
| Actors' Stress Appraisals | -0.04 | 0.03 | -1.26 | 0.210 | 0.08 |
| Partners' Stress Appraisals | 0.02 | 0.03 | 0.65 | 0.516 | 0.04 |
| Actors' Relationship Satisfaction | -0.12 | 0.11 | -1.09 | 0.278 | 0.07 |
| Partners' Relationship Satisfaction | -0.20 | 0.11 | -1.76 | 0.080 | 0.11 |

## Longitudinal Analyses from Studies 2 and 3

As part of our theoretical development and pre-registration for Study 3, we had included a longitudinal component in our analytic plan. However, across Studies 2 and 3, we found no support for the longitudinal component of our model. Thus, we mention this as a key limitation in the general discussion, and refer the reader to this supplemental material for a full report of the analyses that were conducted.

*How partners appraise stress and behave during interactions will have implications for their relationship in long-term.* Stress appraisals and behaviors will then create an upward or downward spiral in the relationship: Over time, these appraisals and behaviors are likely to help attenuate relationship insecurities and foster more satisfying relationships. That is, we view interpersonal stress as a process model (e.g., Ford & Gross, 2019; Gross, 2015), in which the short-term outcomes from one context or conversation could influence future appraisals of stress, behavior within relationship-relevant interactions, as well as have implications for broader patterns of satisfaction and security within a relationship. We focused on relationship insecurities and satisfaction as these are often identified as central outcomes in key relationship theories related to social support, stress, adversity, and trait-like approach and avoidance orientations in relationships (Feeney & Collins, 2015; Gable & Gosnell, 2013; Gable & Impett, 2012; Neff & Karney, 2017; Overall et al., 2022; Reis & Clark, 2013; Rusbult & Van Lange, 2008; Simpson & Rholes, 2017).

***Study 2***

**Long-term outcomes.** When first arriving at the lab session and then six months after completing the lab session, participants completed the Adult Attachment Questionnaire (Simpson et al., 1996). Nine items assessed attachment anxiety (e.g., *I often worry that my romantic partners don’t really love me*) and eight items assessed attachment avoidance (e.g., *I’m not very comfortable having to depend on romantic partners*). Participants responded on 7-point Likert scales (1 = *strongly disagree*, 7 = *strongly agree*). These items were averaged to form anxiety (α = .855, .869) and avoidance (α = .803, .804) composites***.*** The 16-item version of the Couples Satisfaction Index (Funk & Rogge, 2007) was used to assess participants’ general satisfaction in their romantic relationship (α = .939, .964).

**Analyses of stress appraisals and approach and avoidance behaviors on relationship insecurities and satisfaction 6 months later.** Analyses tested the extent to which anticipatory stress appraisals and approach and avoidance behaviors during the conversation predicted changes in relationship security and satisfaction six months later. Actors whose partners engaged in more avoidance behaviors were less avoidantly attached six months later. No other effects were significant (see Table S14).

***Study 3***

**Long-term outcomes.** When first arriving at the lab session and then six months later, participants completed the Adult Attachment Questionnaire (Simpson et al., 1996). Items were averaged to form anxiety (α = .807, .776) and avoidance (α = .768, .782) composites***.*** Five items were used to assess participants’ general satisfaction in their romantic relationship (α = .838, .951).

**Analyses of stress appraisals and approach and avoidance behaviors on relationship insecurities and satisfaction 6 months later.** Analyses tested the extent to which anticipatory stress appraisals and approach and avoidance behaviors during the conversation predicted changes in relationship security and satisfaction six months later. Actors whose partners engaged in more avoidance behaviors more satisfied six months later. No other effects were significant (see Table S15).

| **Table S14** | | | | | | | | | | | | | | | |
| --- | --- | --- | --- | --- | --- | --- | --- | --- | --- | --- | --- | --- | --- | --- | --- |
| *The associations between actors’ and partners’ stress appraisals and approach and avoidance behaviors and long-term outcomes six months later (Study 2)*. | | | | | | | | | | | | | | | |
|  | Anxiety (T2) | | | | | Avoidance (T2) | | | | | Relationship Satisfaction (T2) | | | | |
| Predictor Variables | *B* | *SE* | *t* | *p* | *r* | *B* | *SE* | *t* | *p* | *r* | *B* | *SE* | *t* | *p* | *r* |
| T1 Insecurity/satisfaction | 0.62 | 0.05 | 11.36 | <.001 | 0.63 | 0.66 | 0.05 | 12.61 | <.001 | 0.66 | 0.67 | 0.08 | 8.00 | <.001 | 0.52 |
| Actors' Stress Appraisals | -0.08 | 0.22 | -0.35 | .727 | 0.02 | 0.14 | 0.17 | 0.83 | .410 | 0.06 | -0.43 | 0.32 | -1.33 | .185 | 0.10 |
| Actors' Avoidance Behaviors | 0.43 | 0.50 | 0.86 | .389 | 0.06 | 0.54 | 0.38 | 1.39 | .165 | 0.10 | -1.18 | 0.71 | -1.67 | .097 | 0.12 |
| Actors' Approach Behaviors | 0.30 | 0.66 | 0.45 | .655 | 0.03 | -0.49 | 0.50 | -0.99 | .325 | 0.07 | 0.27 | 0.99 | 0.27 | .784 | 0.02 |
| Partners' Stress Appraisals | -0.13 | 0.22 | -0.61 | .541 | 0.04 | 0.00 | 0.16 | 0.03 | .979 | 0.00 | -0.21 | 0.32 | -0.67 | .502 | 0.05 |
| Partners' Avoidance Behaviors | -0.18 | 0.47 | -0.38 | .701 | 0.03 | -0.97 | 0.35 | -2.79 | .006 | 0.19 | 0.25 | 0.67 | 0.38 | .707 | 0.03 |
| Partners' Approach Behaviors | -1.10 | 0.68 | -1.61 | .109 | 0.11 | -0.08 | 0.51 | -0.16 | .869 | 0.01 | 1.14 | 0.97 | 1.17 | .243 | 0.09 |

| **Table S15** | | | | | | | | | | | | | | | |
| --- | --- | --- | --- | --- | --- | --- | --- | --- | --- | --- | --- | --- | --- | --- | --- |
| *The associations between actors’ and partners’ stress appraisals and approach and avoidance behaviors and long-term outcomes six months later (Study 3)*. | | | | | | | | | | | | | | | |
|  | Anxiety (T2) | | | | | Avoidance (T2) | | | | | Relationship Satisfaction (T2) | | | | |
| Predictor Variables | *B* | *SE* | *t* | *p* | *r* | *B* | *SE* | *t* | *p* | *r* | *B* | *SE* | *t* | *p* | *r* |
| T1 Insecurity/satisfaction | 0.59 | 0.05 | 12.81 | <.001 | 0.64 | 0.62 | 0.05 | 11.92 | <.001 | 0.62 | 0.80 | 0.10 | 7.69 | <.001 | 0.45 |
| Actors' Stress Appraisals | 0.01 | 0.02 | 0.43 | 0.667 | 0.03 | -0.01 | 0.02 | -0.70 | .485 | 0.05 | -0.03 | 0.02 | -1.08 | .282 | 0.07 |
| Actors' Avoidance Behaviors | 0.04 | 0.04 | 1.14 | 0.257 | 0.07 | 0.07 | 0.04 | 1.94 | .053 | 0.13 | 0.04 | 0.05 | 0.84 | .404 | 0.05 |
| Actors' Approach Behaviors | -0.01 | 0.04 | -0.36 | 0.716 | 0.02 | -0.03 | 0.04 | -0.76 | .451 | 0.05 | 0.04 | 0.05 | 0.71 | .476 | 0.05 |
| Partners' Stress Appraisals | 0.02 | 0.02 | 1.13 | 0.260 | 0.07 | 0.01 | 0.02 | 0.32 | .748 | 0.02 | -0.03 | 0.02 | -1.22 | .224 | 0.08 |
| Partners' Avoidance Behaviors | 0.06 | 0.04 | 1.50 | 0.135 | 0.10 | -0.05 | 0.04 | -1.51 | .133 | 0.10 | 0.10 | 0.05 | 2.04 | .043 | 0.13 |
| Partners' Approach Behaviors | 0.00 | 0.04 | 0.00 | 0.996 | 0.00 | 0.01 | 0.04 | 0.34 | .737 | 0.02 | 0.03 | 0.05 | 0.65 | .519 | 0.04 |

# Summary of exploratory analyses that examined potential actor by partner appraisal interaction effects

We re-ran all models reported in the manuscript with the addition of an interaction term between actors’ and partners’ stress appraisals. Across all models and studies, no significant interaction effects were observed. We reference these additional analyses in the ancillary analyses section at the end of each study.

**Summary of exploratory analyses that controlled for stress severity and intensity**

We believe that stress severity or intensity is a subjective indicator—two people experiencing the same stressor frequently report different emotions and experiences. Like other affective states, the presentation of demands/stressors (and subsequent appraisals) are what make situations stressful (e.g., Crum et al., 2020; Epel et al., 2018). Behavioral codes could potentially capture aspects of severity like effort required, time constraints, or evaluative pressure, but ultimately whether an individual is experiencing a stressor as “severe” or “intense” is based on their own interpretations of their situation. Moreover, the BPS model of challenge and threat emphasizes how it is the balance of appraisals of demands relative to resources (and not the severity of the stressor) that most directly determine stress responses regardless of severity.

We went back through our three studies and found items that most closely reflected the construct of “stress severity” or “stress intensity.” In Study 1, participants were asked, “*How stressful do you anticipate the upcoming discussion being?*” from 1 (not at all) to 7 (very much). In Study 2, after the conversation participants were asked the extent to which they agreed that the conversation was intense from –4 (strongly disagree) to 4 (strongly agree). In Study 3, prior to the conversation participants were asked to what extent the issue was a serious problem in their relationship from 1 (not at all) to 7 (extremely serious).

If our claim that what best predicts behavior is the appraisals of resources relative to demands, rather than the overall severity or intensity of stress, controlling for severity/intensity items should not change our original pattern of results despite their significant association with our appraisal measure (*r* ~ -0.5 across the three studies). Tests of moderation were also requested during the review process. Thus, we completed two sets of additional analyses. First, we included two additional main effects of actor and partner self-reported stress severity/intensity to the main analyses. Second, we included the interactions between actor stress severity/intensity and all actor effects as well as partner stress severity/intensity and all partner effects. Across all analyses, the pattern of results was consistent with the original findings. Moreover, there was little evidence of moderation and, in the rare cases where a moderation effect was observed, these moderation effects did not replicate across studies. We mention these additional ancillary analyses within the results section of each study.

# Summary of exploratory analyses that operationalized stress appraisals as resources, demands, and resources × demands

Our *a priori* data analytic plan regarding how to calculate the stress appraisal measure is consistent with BPS research (e.g., Behnke & Kaczmarek, 2018; Beltzer et al., 2014; Gresham et al., 2023; Mendes et al., 2007; Peters et al., 2018; Seery, 2011). While resource and demand appraisals are distinct constructs, the psychological product of stress appraisals (challenge and threat states) are considered anchors of a continuum of stress states (e.g., Blascovich & Mendes, 2010; Jamieson, 2017, Jamieson & Elliot, 2018). Thus, when interpreting research that incorporates physiological and self-report measures, it is common to refer to response states as “more threat and less challenge” and/or “more challenge and less threat” (e.g., Behnke & Kaczmarek, 2018; Beltzer et al., 2014; Gresham et al., 2023; Mendes et al., 2007; Peters et al., 2018; Seery, 2011; Tudder et al., 2023). In the current work, we took an approach that reflects this continuum with higher scores indicating greater challenge and less threat, an approach that has been commonly done in past BPS research when wanting to simplify analyses (e.g., Behnke & Kaczmarek, 2018; Beltzer et al., 2014; Gresham et al., 2023; Mendes et al., 2007; Peters et al., 2018; Seery, 2011).

Moreover, adding resources and demands into the analytical models simultaneously—but separately—raises statistical concerns. Specifically, the shared variance between resources and demands in predicting key outcomes is removed. For example, zero-order correlations across all three studies reveal that greater resources were associated with less demands (S1: *r* = -.198, *p* < .001, S2: *r* = -.525, *p* < .001; S3: r = -.551, *p* < .001).

Thus, for both theoretical and statistical reasons, we opted to retain the original analytic approach. However, to be as responsive and thorough as possible, we explored an alternative analytic approach based on a Reviewer’s comment. To summarize, we entered three fixed effects for actors: 1) demands, 2) resources, 3) and their interaction; and three fixed effects for partners 1) partners’ demands, 2) partners’ resources, 3) and their interaction. For ancillary analyses with experimental condition, role, and relationship satisfaction, we conducted the analyses in the same way we did in the main manuscript in which experimental condition and role were fully crossed with all actor and partner effects (Studies 1 and 2) and actors’ and partners’ relationship satisfaction were entered in as covariates (Studies 1, 2, and 3). Across all three studies, only one interaction between actors’ resource and demand appraisals emerged (Study 1, hypothesis 2). However, this interaction did not remain significant when conducting ancillary analyses with experimental condition and conversation role. Thus, exploratory analyses suggest that we are not missing out on potentially interesting interaction effects between demands and resources.

What about the main effects of resources and demands? Including resources and demands in the models together did not reveal new dynamics to the stress appraisal process that were possibly obfuscated by combining them. As we summarize below, these new analyses across studies were not consistent across studies, nor were they as consistent as the results observed with the original analytic approach.

For H1 (i.e., associations between appraisals and behaviors), approach behaviors were sometimes more strongly predicted by actor demands (Study 1, Study 3), partner demands (Study 2), actor resources (Study 2, Study 3), and/or partner resources (Study 3). Similarly, avoidance behaviors were sometimes more strongly predicted by actor demands (Study 2, Study 3) or partner demands (Study 2).

Exploratory analyses revealed a more consistent pattern for H2 (associations between appraisals and feelings of relationship security and well-being). Support for H2 was generally more strongly predicted by actor resources (Study 1 and 2) and partner resources (Study 1 and 2). However, there were no significant effects observed in Study 3, and the partner resource effect observed in Study 2 did not hold in ancillary analyses.

Support for H3 was generally consistent with the original analyses. That is, adding in resources, demands, and their interaction did not alter the relations between approach and avoidance behaviors and feelings of relationship security and well-being across the three studies.

Taken together, the original analytic approach provided more consistent support for H1 and H2 across the three studies and findings held in ancillary analyses. The greater inconsistencies observed when taking this exploratory analytic approach illustrate that stress appraisals may sometimes be more influenced by demands or resources across different people and situations, but – consistent with the BPS model of challenge and threat – the combined psychological product (challenge and threat continuum) has more consistent and robust predictive power. Given the theoretical and methodological underpinning of our pre-registered approach, as well as the statistical concerns with assessing resources and demands separately, we do not fully report these additional analyses.

# Supplemental Discussion

## Longitudinal Findings

Across Studies 2 and 3, we found little evidence for the potential long-term consequences that appraisals of stress and approach- and avoidance-oriented behavior may have on relationships. When looking at Study 2 in isolation, we thought that the lack of effects were due to attrition and consequently a lack of power to test effects. However, attrition was much lower in Study 3. Additionally, across all three studies, participants were generally college-aged and had been in their relationships for an average of 1-4 years, depending on the sample. Thus, there could have been potential ceiling effects as we generally had satisfied couples participating in our studies. We still believe there is potential merit to the idea that stress appraisals and approach- and avoidance-oriented behavior could contribute to an upward or downward cycle in relationships, contributing to changes in relationship satisfaction and securities. We speculate that perhaps six months was not the correct timeframe to examine these longitudinal changes and that our sample of young, relatively satisfied couples undermined our tests of this hypothesis. We encourage longitudinal, prospective work to continue potential longitudinal effects of stress appraisals and approach- and avoidance-oriented behaviors within interactions.

**References**

Behnke, M., & Kaczmarek, L. D. (2018). Successful performance and cardiovascular markers of challenge and threat: A meta-analysis. *International Journal of Psychophysiology*, *130*, 73–79. https://doi.org/10.1016/j.ijpsycho.2018.04.007

Beltzer, M. L., Nock, M. K., Peters, B. J., & Jamieson, J. P. (2014). Rethinking butterflies: The affective, physiological, and performance effects of reappraising arousal during social evaluation. *Emotion*, *14*(4), 761–768. https://doi.org/10.1037/a0036326

Crum, A. J., Jamieson, J. P., & Akinola, M. (2020). Optimizing stress: An integrated intervention for regulating stress responses. *Emotion*, *20*(1), 120–125. https://doi.org/10.1037/emo0000670

Epel, E. S., Crosswell, A. D., Mayer, S. E., Prather, A. A., Slavich, G. M., Puterman, E., & Mendes, W. B. (2018). More than a feeling: A unified view of stress measurement for population science. *Frontiers in Neuroendocrinology*, *49*, 146–169. https://doi.org/10.1016/j.yfrne.2018.03.001

Feeney, B. C., & Collins, N. L. (2015). A new look at social support: A theoretical perspective on thriving through relationships. *Personality and Social Psychology Review*, *19*, 113–147. https://doi.org/10.1177/1088868314544222

Ford, B. Q., & Gross, J. J. (2019). Why beliefs about emotion matter: An emotion-regulation perspective. *Current Directions in Psychological Science*, *28*, 74–81. https://doi.org/10.1177/0963721418806697

Gable, S. L., & Gosnell, C. L. (2013). Approach and avoidance behavior in interpersonal relationships. *Emotion Review*, *5*, 269–274. https://doi.org/10.1177/1754073913477513

Gable, S. L., & Impett, E. A. (2012). Approach and avoidance motives and close relationships. *Social & Personality Psychology Compass*, *6*(1), 95–108. https://doi.org/10.1111/j.1751-9004.2011.00405.x

Gresham, A. M., Peters, B. J., Tudder, A., & Simpson, J. A. (2023). Sense of power and markers of challenge and threat during extra-dyadic problem discussions with romantic partners. *Psychophysiology*, *60*(11), e14379. https://doi.org/10.1111/psyp.14379

Gross, J. J. (2015). Emotion regulation: Current status and future prospects. *Psychological Inquiry*, *26*(1), 1–26. https://doi.org/10.1080/1047840X.2014.940781

Jamieson, J. P. (2017). Challenge and threat appraisals. *Handbook of Competence and Motivation*: *Theory and application*. 2, 175-191.

Jamieson, J. P., & Elliot, A. J. (2018). To Approach or to Avoid: Integrating the Biopsychosocial Model of Challenge and Threat with Theories from Affective Dynamics and Motivation Science. In G. Oettingen, A. T. Sevincer, & P. M. Gollwitzer (Eds.), *The Psychology of Thinking about the Future* (pp. 440–454). The Guilford Press.

Jamieson, J. P., Nock, M. K., & Mendes, W. B. (2012). Mind over matter: Reappraising arousal improves cardiovascular and cognitive responses to stress. *Journal of Experimental Psychology*, *141*(3), 417–422. https://doi.org/10.1037/a0025719

Mendes, W. B., Blascovich, J., Hunter, S. B., Lickel, B., & Jost, J. T. (2007). Threatened by the unexpected: Physiological responses during social interactions with expectancy-violating partners. *Journal of Personality and Social Psychology*, *92*(4), 698–716. https://doi.org/10.1037/0022-3514.92.4.698

Mendes, W. B., Blascovich, J., Major, B., & Seery, M. (2001). Challenge and threat responses during downward and upward social comparisons. *European Journal of Social Psychology*, *31*(5), 477–497. https://doi.org/10.1002/ejsp.80

Neff, L. A., & Karney, B. R. (2017). Acknowledging the elephant in the room: How stressful environmental contexts shape relationship dynamics. *Current Opinion in Psychology*, *13*, 107–110. https://doi.org/10.1016/j.copsyc.2016.05.013

Overall, N. C., Pietromonaco, P. R., & Simpson, J. A. (2022). Buffering and spillover of adult attachment insecurity in couple and family relationships. *Nature Reviews Psychology*, *1*(2), Article 2. https://doi.org/10.1038/s44159-021-00011-1

Peters, B. J. (2017). *The consequences of having an insecure partner: A pre-emptive buffering model* [Doctoral Dissertation, University of Rochester]. http://hdl.handle.net/1802/33190

Peters, B. J., & Jamieson, J. P. (2016). The consequences of suppressing affective displays in romantic relationships: A challenge and threat perspective. *Emotion*, *16*(7), 1050–1066. https://doi.org/10.1037/emo0000202

Peters, B. J., Overall, N. C., Girme, Y. U., & Jamieson, J. P. (2019). Partners’ attachment insecurity predicts greater physiological threat in anticipation of attachment-relevant interactions. *Journal of Social and Personal Relationships*, *36*(2), 469–489. https://doi.org/10.1177/0265407517734655

Peters, B. J., Overall, N. C., & Jamieson, J. P. (2014). Physiological and cognitive consequences of suppressing and expressing emotion in dyadic interactions. *International Journal of Psychophysiology*, *94*(1), 100–107. https://doi.org/10.1016/j.ijpsycho.2014.07.015

Peters, B. J., Reis, H. T., & Jamieson, J. P. (2018). Cardiovascular indexes of threat impair responsiveness in situations of conflicting interests. *International Journal of Psychophysiology*, *123*, 1–7. https://doi.org/10.1016/j.ijpsycho.2017.12.005

Reis, H. T., & Clark, M. S. (2013). Responsiveness. In *The Oxford handbook of close relationships* (pp. 400–423). Oxford University Press. https://doi.org/10.1093/oxfordhb/9780195398694.001.0001

Rusbult, C. E., & Van Lange, P. A. M. (2008). Why we need interdependence theory. *Social and Personality Psychology Compass*, *2*, 2049–2070. https://doi.org/10.1111/j.1751-9004.2008.00147.x

Seery, M. D. (2011). Challenge or threat? Cardiovascular indexes of resilience and vulnerability to potential stress in humans. *Neuroscience and Biobehavioral Reviews*, *35*(7), 1603–1610. https://doi.org/10.1016/j.neubiorev.2011.03.003

Simpson, J. A., & Rholes, W. S. (2017). Adult attachment, stress, and romantic relationships. *Current Opinion in Psychology*, *13*, 19–24. https://doi.org/10.1016/j.copsyc.2016.04.006

Simpson, J. A., Rholes, W. S., & Phillips, D. (1996). Conflict in close relationships: An attachment perspective. *Journal of Personality and Social Psychology*, *71*, 899–914. https://doi.org/10.1037/0022-3514.71.5.899

Tomlinson, J. M., Feeney, B. C., Peters, B. J., & Zhang, Y. (2022). Physiological correlates of support for self-expansion and links to goal pursuit in retirement. *Psychophysiology*, *59*(9), e14061. https://doi.org/10.1111/psyp.14061

Tudder, A., Gresham, A. M., Peters, B. J., Reis, H. T., & Jamieson, J. P. (2020). The effects of dispositional restrictiveness on physiological markers of challenge and threat during a hypothetical transitional period in romantic relationships. *Psychophysiology*, *57*(10), e13624. https://doi.org/10.1111/psyp.13624

Tudder, A., Wilkinson, M., Gresham, A. M., & Peters, B. J. (2023). The intrapersonal and interpersonal consequences of a new experimental manipulation of co-rumination. *Emotion*, *23*, 1190–1201. https://doi.org/10.1037/emo0001151

Weissman, D. G., & Mendes, W. B. (2021). Correlation of sympathetic and parasympathetic nervous system activity during rest and acute stress tasks. *International Journal of Psychophysiology*, *162*, 60–68. https://doi.org/10.1016/j.ijpsycho.2021.01.015
